# PEPD is a pivotal regulator of p53 tumor suppressor

**DOI:** 10.1038/s41467-017-02097-9

**Published:** 2017-12-12

**Authors:** Lu Yang, Yun Li, Arup Bhattacharya, Yuesheng Zhang

**Affiliations:** 1 Department of Pharmacology and Therapeutics, Roswell Park Cancer Institute, Buffalo, NY 14263 USA; 2 Department of Urology, Roswell Park Cancer Institute, Buffalo, NY 14263 USA; 3 Department of Cancer Prevention and Control, Roswell Park Cancer Institute, Buffalo, NY 14263 USA

## Abstract

p53 tumor suppressor responds to various cellular stresses and regulates cell fate. Here, we show that peptidase D (PEPD) binds and suppresses over half of nuclear and cytoplasmic p53 under normal conditions, independent of its enzymatic activity. Eliminating PEPD causes cell death and tumor regression due to p53 activation. PEPD binds to the proline-rich domain in p53, which inhibits phosphorylation of nuclear p53 and MDM2-mediated mitochondrial translocation of nuclear and cytoplasmic p53. However, the PEPD-p53 complex is critical for p53 response to stress, as stress signals doxorubicin and H_2_O_2_ each must free p53 from PEPD in order to achieve robust p53 activation, which is mediated by reactive oxygen species. Thus, PEPD stores p53 for the stress response, but this also renders cells dependent on PEPD for survival, as it suppresses p53. This finding provides further understanding of p53 regulation and may have significant implications for the treatment of cancer and other diseases.

## Abstract (web summary)

p53 is a pivotal tumour suppressor that is activated by various cellular stress inducers. Here, the authors show that peptidase D (PEPD) promotes the growth of cancer cells by suppressing p53 and that the complex PEPD-p53 is critical for robust p53 activation in response to stress signals.

## Introduction

Peptidase D (PEPD), also known as prolidase among other names, was discovered 80 years ago to hydrolyze dipeptides with proline or hydroxyproline at the carboxy terminus^1^. It is expressed ubiquitously and important for collagen metabolism^2,3^. PEPD also upregulates hypoxia-inducible factor-1, transforming growth factor beta 1 and its receptor via its catalytic products^4,5^. Loss of enzymatic activity, due to PEPD gene mutation, is widely believed to be responsible for a disease known as PEPD deficiency (PD), which involves multiple organs and tissues, e.g., skin ulcer, reduced bone growth, splenomegaly, immune malfunction, and mental retardation^2,6^. However, therapies aimed at ameliorating PEPD enzymatic loss or enhancing collagen metabolism are largely ineffective^2,7^. PD remains incurable.

We recently found that PEPD is a ligand of ErbB1 and ErbB2 which are oncogenic receptor tyrosine kinases, that the enzymatic function of PEPD is not needed for this activity, and that intracellular PEPD has no effect on these receptors^8–10^. It remains unclear about the physiological importance of PEPD as a ligand of ErbB1 and ErbB2 or the involvement of these receptors in PD, as circulating PEPD level is kept low by a plasma proteolysis pathway^11^. However, recombinant human PEPD or an enzymatically inactive mutant, when added to cell culture or injected to tumor-bearing mice (with inhibition of the plasma proteolysis pathway), strongly inhibits the growth of cancer cells overexpressing ErbB1 and/or ErbB2^9,10,12^. Thus, recombinant PEPD or its mutant is a promising cancer therapeutic. In addition, PEPD modulates expression of interferon α/β receptor IFNAR1, which is also independent of PEPD enzymatic activity^13^. These findings reveal the hidden but important functions of PEPD.

We now present data showing that PEPD also suppresses p53, a pivotal multifunctional tumor suppressor^14^. p53 regulation has been extensively studied^15^, but we find that PEPD directly binds to p53 in the nucleus and cytoplasm and suppresses both transcription-dependent and transcription-independent activities of p53, which does not require PEPD enzymatic activity. We further find that PEPD suppression of p53 is essential for cell survival and tumor growth. p53 is activated by various cellular stress inducers. Using doxorubicin (DOX) and hydrogen peroxide (H_2_O_2_) as examples, we find that the PEPD-p53 complex serves as a p53 depot which enables robust p53 activation by stress. These findings uncover an important physiological function of PEPD and a critical new regulatory mechanism of p53.

## Results

### PEPD loss leads to cell death and tumor regression

**Fig. 1:**
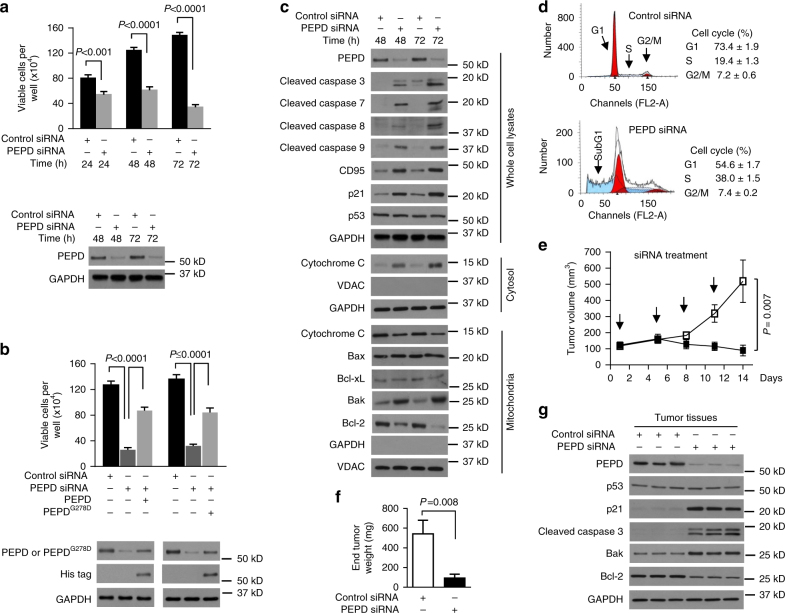
PEPD is essential for cell survival in vitro and in vivo. **a** Measurement of UM-UC-3 cell viability and IB analysis of PEPD after siRNA treatment. **b** Measurement of UM-UC-3 cell viability and IB analysis of PEPD after siRNA treatment for 24 h and then treatment with or without PEPD or PEPD^G278D^ (His-tagged) for 48 h. **c** IB analysis of various proteins in UM-UC-3 cells after siRNA treatment. **d** UM-UC-3 cell cycle analysis by flow cytometry after siRNA treatment for 72 h. **e**, **f** UM-UC-3 tumor growth in athymic nude mice after intratumoral injection of control siRNA (open square) or PEPD siRNA (filled square) at the indicated times (arrows) and final tumor weight. **g** IB analysis of various proteins in tumors from 3 mice/group obtained on day 14. GAPDH and VDAC were measured to ensure the purity of subcellular fractions or as loading controls. Cells were cultured in 6-well plates (5 × 10^4^ cells/well) for 24 h before experimental treatment in **a**–**d.** Data are means ± s.d. (*n* = 3) in **a**, **b**, and **d** (two-way ANOVA followed by Tukey multiple comparisons test) and means ± s.e.m. (*n* = 8) in **e** and **f** (two-tailed *t*-test)

Our PEPD investigation began with a commonly used human bladder cancer cell line, UM-UC-3, which was established from a transitional cell carcinoma^16^. PEPD knockout by CRISPR/Cas9 led to rapid and total killing of UM-UC-3 cells (Supplementary Fig. 1). Same results were obtained using normal human urothelial cells and immortalized human urothelial cells (Supplementary Figs. 2 and 3). A PEPD siRNA also caused marked decrease in PEPD expression in UM-UC-3 cells and progressive decrease in cell survival, reaching ~78% cell death at 72 h (Fig. 1a; Supplementary Fig. 4a). However, overexpressing PEPD in UM-UC-3 cells did not impact cell growth (Supplementary Fig. 4b, c). Cell death caused by PEPD siRNA could be partially prevented by adding to the culture medium either recombinant human PEPD or a mutant (PEPD^G278D^), both of which entered cells and partially prevented PEPD loss (Fig. 1b). Thus, cell death caused by PEPD siRNA is not due to an off-target effect. Because PEPD^G278D^ is enzymatically inactive^17^, the above result also indicates that cell death caused by PEPD knockdown is not due to loss of PEPD enzymatic activity. Indeed, neither glycylproline nor proline (the enzymatic substrate and product of PEPD, respectively) impacted cell survival or rescued cells from death caused by PEPD siRNA (Supplementary Fig. 4d, f).

Because PEPD is a ligand of ErbB1 and ErbB2, we next turned to murine myeloid 32D cells, which express neither ErbB1 nor ErbB2^18^. PEPD knockdown in 32D cells also caused cell death (Supplementary Fig. 4g, h), indicating that cell death due to PEPD loss does not involve ErbB signaling. Indeed, we previously showed that intracellular PEPD has no effect on ErbB1 and ErbB2^8,9^.

Cells with PEPD knockdown by siRNA showed activation of both intrinsic and extrinsic apoptosis pathways: upregulation of Bak and Bax and downregulation of Bcl-2 in the mitochondria, cytochrome C release from the mitochondria to the cytosol, and activation of caspases 3, 7, and 9, as well as upregulation of Fas receptor (CD95) and activation of caspase 8 (Fig. 1c). Moreover, these cells also showed upregulation of p21, a key negative regulator of G1 and S phases^19^, and S phase arrest (Fig. 1c, d). These results suggest p53 activation, as CD95, p21, Bak, Bax, and Bcl-2 are well known transcriptional targets of p53. However, neither p53 nor Bcl-xL (a p53 target) responded to PEPD knockdown (Fig. 1c).

To assess the role of PEPD in vivo, we generated subcutaneous tumors in nude mice using UM-UC-3 cells and performed intratumoral injection of siRNA once every 3–4 days for four times. Tumors grew rapidly on control siRNA, but PEPD siRNA caused progressive tumor regression; at the end of the experiment, average tumor size and tumor weight in the PEPD siRNA group were only 17.3% and 17.1% of that in the control siRNA group (Fig. 1e, f). PEPD siRNA markedly downregulated PEPD and Bcl-2, markedly upregulated p21 and Bak, and caused strong activation of caspase 3, but did not alter p53 expression level in the tumors (Fig. 1g), which are very similar to that in cultured UM-UC-3 cells. These results provide in vivo evidence that PEPD is essential for cell survival.

### PEPD protects cells by suppressing p53

**Fig. 2:**
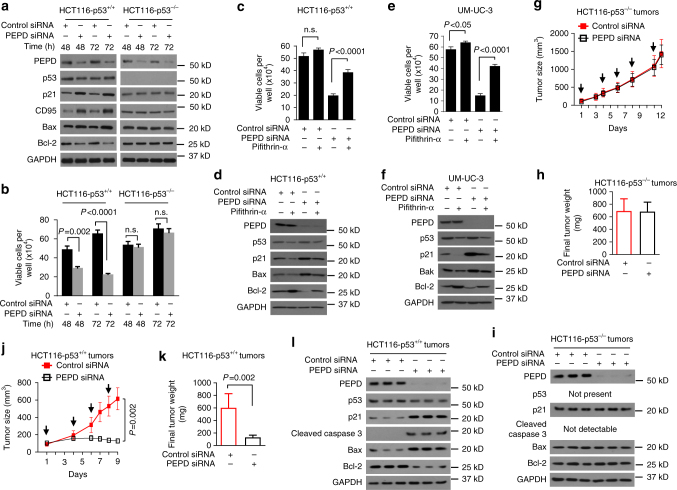
PEPD protects cells by suppressing p53. **a**, **b** IB analysis of various proteins and measurement of cell viability after siRNA treatment. **c**–**f** Measurement of cell viability and IB analysis of proteins after treatment with siRNA in the absence or presence of pifithrin-α (30 μM) for 72 h. **g**–**i** HCT116-p53^−/−^ tumor growth in athymic nude mice after intratumoral injection of control siRNA or PEPD siRNA at the indicated times (arrows), final tumor weight, and IB analysis of various proteins in 3 tumors per group obtained on day 12. **j**–**l** HCT116-p53^+/+^ tumor growth in athymic nude mice after intratumoral injection of control siRNA or PEPD siRNA at the indicated times (arrows), final tumor weight, and IB analysis of various proteins in 3 tumors per group obtained on day 9. Cells were grown in 12-well plates (2 × 10^4^ cells/well) for 24 h before experimental treatment in **a**–**f**. Data are means ± s.d. (*n* = 3) in **b**, **c** and **e** (two-way ANOVA followed by Tukey multiple comparisons test) and means ± s.e.m. (*n* = 7–8) in **g**, **h**, **j**, and **k** (two-tailed *t*-test); n.s., not significant. GAPDH was used as a loading control in the IB experiments

To understand the role of p53 in cell death caused by PEPD knockdown, we turned to human colon cancer HCT116 cells with and without p53^20^. PEPD levels are similar between the two cell lines (Supplementary Fig. 5a) and were similarly reduced by PEPD siRNA (Fig. 2a). As in UM-UC-3 cells, PEPD knockdown did not alter p53 level but apparently activated p53 in HCT116-p53^+/+^ cells (increased expression of p21, CD95 and Bax, and decreased expression of Bcl-2), since PEPD knockdown had no effect on the p53 targets in HCT116-p53^−/−^ cells (Fig. 2a). More importantly, whereas the survival of HCT116-p53^+/+^ cells decreased markedly and time-dependently upon PEPD siRNA treatment, the survival of HCT116-p53^−/−^ cells was not affected by PEPD knockdown (Fig. 2b). Pifithrin-α, a p53 inhibitor^21^, could also rescue both HCT116-p53^+/+^ cells and UM-UC-3 cells against PEPD knockdown. Cells were treated with siRNA (72 h) with or without pifithrin-α at optimal concentration (30 μM). With control siRNA, pifithrin-α slightly increased cell growth, but with PEPD siRNA, pifithrin-α increased cell survival by 96–184% (Fig. 2c and e), which was accompanied by inhibition of p53 signaling (decreased expression of p21 and Bax, and increased expression of Bcl-2) (Fig. 2d and f). Notably, pifithrin-α downregulates p53, regardless of PEPD knockdown (Fig. 2d and f), the reason for which is unknown, but it was also seen in other cells^22^, and additional activities of pifithrin-α were reported^23^. Moreover, a pan-caspase inhibitor (Z-VAD-FMK) also strongly rescued cells against PEPD knockdown, increasing the survival of HCT116-p53^+/+^ cells and UM-UC-3 cells against PEPD siRNA (72 h treatment) by 233.9–296.7% (Supplementary Fig. 5b, c). Finally, we compared the response of HCT116-p53^+/+^ tumors and HCT116-p53^−/−^ tumors to PEPD knockdown. We generated subcutaneous tumors in nude mice. HCT116-p53^−/−^ tumors grew faster than HCT116-p53^+/+^ tumors (Fig. 2g, j), reflecting the tumor-suppressing activity of p53, although the latter tumors seem heavier (Fig. 2h, k). Intratumor injection of PEPD siRNA once every 2–3 days strongly downregulated PEPD in both types of tumors, strongly inhibited HCT116-p53^+/+^ tumors, which was accompanied by p53 activation (Bcl-2 downregulation, upregulation of p21 and Bax, and caspase 3 activation) in the tumors, but had no effect on HCT116-p53^−/−^ tumor growth or the p53 targets in these tumors (Fig. 2g–l).

Collectively, our results show that PEPD suppression of p53 is essential for cell survival and tumor growth. Notably, p53 in UM-UC-3 cells carries a mutation (F113C)^24^, but apparently remains functional, whereas HCT116-p53^+/+^ cells carry wild-type (WT) p53^20^.

### PEPD inhibits transcription-independent function of p53

**Fig. 3:**
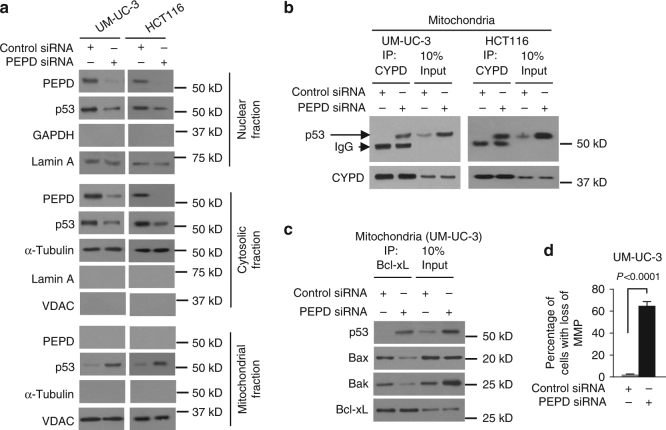
PEPD inhibits transcription-independent p53 activity. **a** IB analysis of p53 relocation in cells after siRNA treatment for 72 h. Lamin A, α-tubulin and VDAC were measured to ensure the purity of the subcellular fractions or as loading controls. **b** IB analysis of p53 binding to CYPD in mitochondria after cell treatment with siRNA for 72 h. **c** IB analysis of Bcl-xL binding to p53, Bax and Bak in mitochondria after cell treatment with siRNA for 72 h. **d** Flow cytometry analysis of MMP after cell treatment with siRNA for 72 h. Cells were cultured in 6-well plates (5–10 × 10^4^ cells/well) for 24 h before experimental treatment. Data are means ± s.d. (*n* = 3)

To understand how PEPD knockdown activates p53 without altering its expression, we measured subcellular distribution of p53 in UM-UC-3 cells and HCT116 (p53^+/+^) cells. PEPD is present in both nucleus and cytosol but absent in mitochondria, and PEPD siRNA knocked down both nuclear and cytosolic PEPD in both cell lines (Fig. 3a; Supplementary Fig. 6a). PEPD knockdown caused nuclear and cytosolic p53 to translocate to the mitochondria in both cell lines (Fig. 3a). p53 accumulation in the mitochondrial matrix triggers mitochondrial permeability transition pore opening and necrosis by physical interaction with cyclophilin D (CYPD)^25^. Indeed, mitochondrial p53 accumulation, in response to PEPD knockdown, resulted in p53 binding to CYPD in both cell lines (Fig. 3b). Mitochondrial p53 also binds to Bcl-2 and Bcl-xL, neutralizing their inhibitory effects on proapoptotic Bak and Bax^26^. We focused on Bcl-xL in UM-UC-3 cells, taking advantage of the finding that Bcl-xL level does not change following PEPD knockdown in these cells (Fig. 1c). We found that PEPD knockdown-induced mitochondrial p53 accumulation is accompanied by increased p53 association with Bcl-xL and disruption of Bcl-xL association with Bax and Bak (Fig. 3c). Moreover, PEPD knockdown caused marked increase in cells with loss of mitochondrial transmembrane potential (MMP) (Fig. 3d, Supplementary Fig. 6b). Thus, PEPD prevents both nuclear and cytosolic p53 from moving to mitochondria to initiate apoptosis and necrosis.

### PEPD inhibits p53 transcriptional activity

**Fig. 4:**
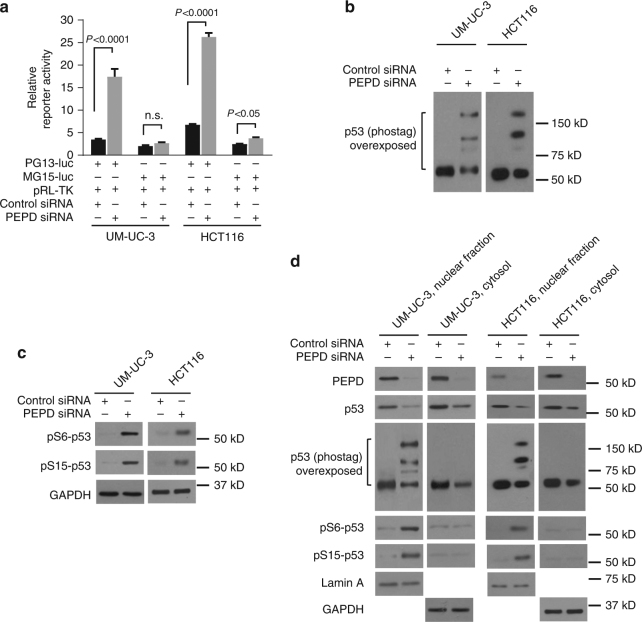
PEPD inhibits transcription function of p53. **a** Reporter activity in cells after plasmid transfection for 24 h and then treatment with siRNA for 48 h. **b**, **c** Phos-tag IB analysis and IB analysis of p53 phosphorylation in cells treated with siRNA for 72 h. **d** IB analysis of various proteins in nuclear fraction and cytosol of cells treated with siRNA for 72 h. Lamin A and GAPDH were measured as loading controls. Cells were cultured in 6-well plates (5–10 × 10^4^ cells/well) for 24 h before experimental treatment. Data are means ± s.d. (*n* = 3) in **a**; two-way ANOVA followed by Tukey multiple comparisons test; n.s., not significant

We next analyzed the effect of PEPD knockdown on transcriptional function of p53. UM-UC-3 cells and HCT116 cells (WT p53) were transfected with an equal amount of p53 reporter PG13-luc which contains multiple copies of p53-binding sequence or MG15-luc which contains multiple copies of mutated p53-binding sequence^27^, along with pRL-TK (*Renilla* luc) for control of transfection efficiency; 24 h later, cells were treated with control or PEPD siRNA for 48 h. PG13-luc responded to PEPD knockdown by increasing luciferase (luc) expression 5.0-fold in UM-UC-3 cells and 3.9-fold in HCT116 cells, whereas luc expression by MG15-luc increased only slightly in both cell lines following PEPD knockdown (Fig. 4a). Thus, PEPD knockdown markedly increases the transactivation activity of p53, which is consistent with modulation of various p53 target proteins by PEPD siRNA in a p53-dependent manner as described before. Moreover, in both cells lines, PEPD knockdown stimulated p53 phosphorylation (Fig. 4b). Two phosphorylation sites in the p53 transactivation domain (serine 6 and serine 15) were measured, and PEPD knockdown caused phosphorylation at both sites in both cell lines (Fig. 4c). Hence, PEPD inhibits phosphorylation in p53 transactivation domain. By analyzing nuclear fraction and cytosolic fraction separately, we found that PEPD knockdown-induced p53 phosphorylation occurs in nuclear p53 but not cytosolic p53 (Fig. 4d). Collectively, our results show that PEPD suppresses p53 transcriptional activity by inhibiting nuclear p53 phosphorylation in its transactivation domain.

### PEPD binds to over 50% of cellular p53

**Fig. 5:**
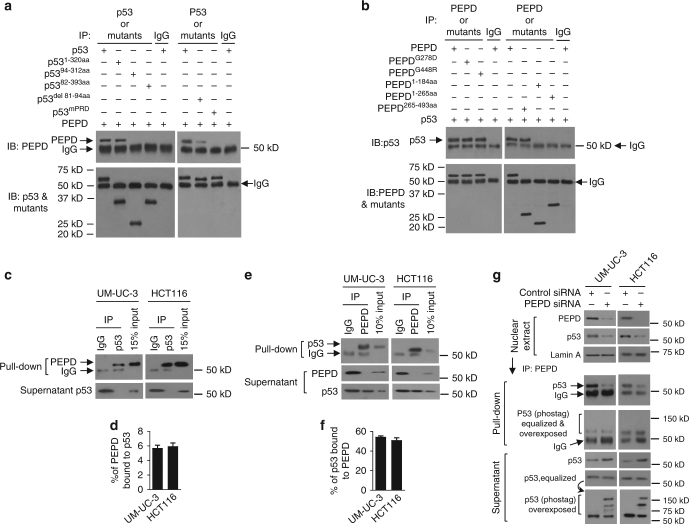
PEPD binds to PRD in p53 and blocks nuclear p53 activation. **a** IP-IB analysis of direct binding of p53 and its mutants to PEPD. **b** IP-IB analysis of direct binding of PEPD and its mutants to p53. **c**–**f** IP-IB analysis of percentages of cellular p53 that binds to PEPD and cellular PEPD that binds to p53. IB bands were quantified by ImageJ. Error bars are mean ± SD (*n* = 3). **g** IB analysis of PEPD and p53 and IP-IB analyses of PEPD-free p53 and phosphor-p53 in nuclear fraction of cells treated with siRNA for 72 h. Lamin A was measured as a loading control. Cells were cultured in 6-well plates (5–10 × 10^4^ cells/well) for 24 h before siRNA treatment

PEPD directly binds to recombinant human p53 (Fig. 5a). p53 is a multi-domain protein, with each subunit of human p53 composed of 393 amino acids (aa). Several mutants of human p53 were evaluated for binding to PEPD, including p53^1–320aa^ (N-terminal 320 aa), p53^94–312aa^ (aa 94–312), p53^82–393aa^ (aa 82–393), p53^del81–94aa^ (deletion of aa 81–94), and p53^mPRD^ (converting 11 Ps to 11As in the proline-rich domain [PRD]) (Supplementary Figs 7a, b). PEPD bound to p53^1–320aa^ as well as it did to WT p53, but failed to bind to p53^94–312aa^ and p53^82–393aa^ (Fig. 5a). Partial deletion of the PRD (p53^del81–94aa^) markedly reduced PEPD binding, whereas p53^mPRD^ could not bind to PEPD at all (Fig. 5a). Thus, PEPD directly binds to PRD in p53, and most of the PRD sequence, if not its entirety, is involved in PEPD binding. Our results also suggest that PEPD may bind to certain p53 mutants.

PEPD is a homodimeric protein, with each subunit of human PEPD composed of 493 aa, containing the N-terminal regulatory domain (1–174 aa), a linker (175–185 aa) and the C-terminal catalytic domain (186-493 aa)^28,29^. Several human PEPD mutants were evaluated for binding to human p53, including PEPD^G278D^, PEPD^G448R^, PEPD^1–184aa^ (N-terminal 184 aa), PEPD^1–265aa^ (N-terminal 265 aa), and PEPD^265–493aa^ (C-terminal 229 aa) (Supplementary Fig. 7c, d). Both PEPD^G278D^ and PEPD^G448R^, despite carrying a single aa change, are enzymatically inactive^17^, but bound to p53, as well as did WT PEPD (Fig. 5b). PEPD^1–184aa^ and PEPD^1–265aa^ failed to bind to p53, but PEPD^265–493aa^ retains full p53 binding ability (Fig. 5b). Notably, PEPD^265–493aa^ cannot form homodimers^9^. Thus, the catalytic domain of PEPD binds to p53, although the enzymatic function of PEPD is not involved in p53 binding and regulation. Our results also suggest that each PEPD monomer may bind to a p53 subunit. SH3 domain, WW domain and EVH1 domain are known to bind to proline-rich motifs^30–32^, but, PEPD^265–493aa^ does not appear to carry any of these domains.

To estimate the percentage of cellular PEPD that binds to p53 in UM-UC-3 cells and HCT116 cells, all p53 molecules in cell lysates were pulled down with a p53 antibody in excess, and the percentage of PEPD molecules that came down with p53 was determined by comparing the intensity of the PEPD band with that of the input control (Fig. 5c; Supplementary Fig. 8a). We found that only about 6% of cellular PEPD molecules are bound to p53 in these cells (Fig. 5d). To estimate the percentage of cellular p53 molecules that bind to PEPD, all PEPD molecules in cell lysates were pulled down with a PEPD antibody in excess, and the percentage of p53 molecules that remained in the supernatant was determined by comparing the intensity of the p53 band with that of the input control (Fig. 5e; Supplementary Fig. 8b). We also compared p53 level in isotype-matched IgG-treated samples and other samples (Fig. 5e) or glyceraldehyde 3-phosphate dehydrogenase (GAPDH) level in all samples (Supplementary Fig. 8b) to confirm the validity of the approach. We found that ~ 51–55% of p53 molecules in the cell lines are bound to PEPD (Fig. 5f). Nearly identical result was obtained by calculating the percentage of p53 present in the pull-down samples. Thus, whereas only a small fraction of cellular PEPD binds to p53, more than half of cellular p53 are bound to PEPD. p53 binds to PEPD in both cytoplasm and nucleus (Supplementary Fig. 8c). Whether some of the binding between p53 and PEPD in cell is indirect remains to be determined, given that p53 binds to other proteins^33^.

### PEPD sequesters nuclear p53 and blocks its phosphorylation

PEPD knockdown results in translocation of nuclear and cytosolic p53 to mitochondria (Fig. 3a). Paradoxically, p53 trans-activation/trans-suppression activity increased upon PEPD knockdown (e.g., Figs. 1c, 4a). To better understand how PEPD inhibits nuclear p53, we treated UM-UC-3 cells and HCT116 cells with control or PEPD siRNA, and then analyzed nuclear p53. As expected, PEPD knockdown resulted in marked decrease in nuclear p53 level (Fig. 5g). Next, PEPD-bound p53 in the nuclear extracts were removed by PEPD pull-down. PEPD knockdown caused no phosphorylation of p53 that remained bound to PEPD (pull-down fraction) (Fig. 5g). However, despite p53 exit from the nucleus due to PEPD knockdown, PEPD-free nuclear p53 level (supernatant fraction) increased significantly in such cells (Fig. 5g). Moreover, it is the PEPD-free nuclear p53 whose phosphorylation increased upon PEPD knockdown (Fig. 5g). Thus, once freed from PEPD, p53 is phosphorylated.

### p53^mPRD^ is inactive

**Fig. 6:**
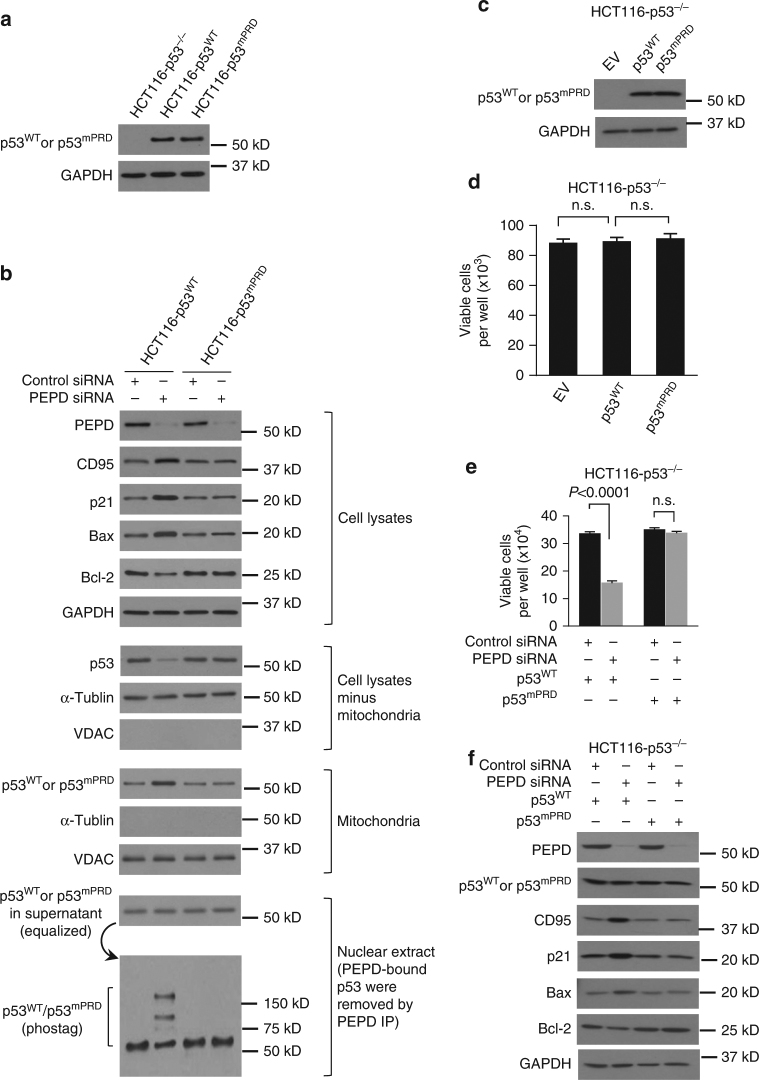
PRD mutation inactivates p53. **a** IB analysis of p53^WT^ and p53^mPRD^. **b** IB analysis of various proteins in whole lysates and subcellular fractions of cells treated with control siRNA or PEPD siRNA for 48 h. **c**, **d** IB analysis of p53^WT^ and p53^mPRD^, as well as analysis of cell viability at 48 h after plasmid transfection. Cells were cultured in 24-well plates (5 × 10^3^ cells/well) for 24 h before plasmid transfection. **e**, **f** Analysis of cell viability and IB analysis of various proteins in cells treated with siRNA for 48 h and then transfected with the plasmid for 48 h. GAPDH, α-tubulin and VDAC were measured to ensure the purity of the subcellular fractions or as loading controls. Cells were cultured in 6-well plates (8 × 10^3^ cells/well) for 24 h before experimental treatment. Data are means ± s.d. (*n* = 3); one-way ANOVA followed by Tukey multiple comparisons test in **d**; two-way ANOVA followed by Tukey multiple comparisons test in **e**; n.s., not significant

Because p53^mPRD^ does not bind to PEPD (Fig. 5a), we wondered whether introducing it to cell might kill the cell, since PEPD would not be able to inhibit it. However, we could stably express in HCT116-p53^−/−^ cells either WT p53 (HCT116-p53^WT^ cells) or p53^mPRD^ (HCT116-p53^mPRD^ cells) at similar levels (Fig. 6a). There was no increase in p53 activity in HCT116-p53^mPRD^ cells regardless of PEPD knockdown, while HCT116-p53^WT^ cells responded to PEPD loss as expected (Fig. 6b). Because the stable clones were generated under selection pressure which could potentially elicit adaptive changes, we also transiently expressed p53^WT^ or p53^mPRD^ in HCT116-p53^−/−^ cells, but neither protein had any effect on cell survival (Fig. 6c, d). However, if the cells were pre-depleted of PEPD by siRNA, p53^WT^ transfection caused cell death resulting apparently from p53 activation, whereas p53^mPRD^ transfection still had no effect (Fig. 6e, f). These results show that p53^mPRD^ is inactive, indicating that the PRD is critical for p53 function. It appears that p53^mPRD^ inactivity results from the mutation, rather than its inability to bind to PEPD.

### PEPD halts MDM2-directed p53 translocation to mitochondria

**Fig. 7:**
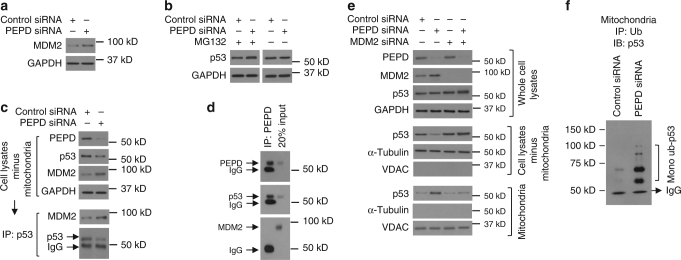
PEPD blocks MDM2-mediated p53 move to mitochondria. **a** IB analysis of MDM2 in cells treated with siRNA for 72 h. **b** IB analysis of p53 in cells treated with siRNA and 24 h later treated with or without MG132 (25 μM) for 48 h. **c** IB and IP-IB analyses of p53 association with MDM2 after cell treatment with siRNA for 48 h. **d** IP-IB analysis of MDM2 binding to the p53-PEPD complex. **e** IB analysis of mitochondria translocation of p53 in cells treated first with control siRNA or MDM2 siRNA and 24 h later treated with control siRNA or PEPD siRNA for 48 h. MDM2 siRNA #1 was used (see Methods). **f** IB analysis of mitochondrial monoubiquitinated p53 in cells treated with siRNA for 48 h, with UbAl (100 μM) added in the final 4 h. GAPDH, α-tubulin and VDAC were measured to ensure the purity of subcellular fractions or as loading controls. Cells were cultured in 6-well plates (5–10 × 10^4^ cells/well) for 24 h before experimental treatment

MDM2 is another essential regulator of p53. MDM2 ubiquitinates and antagonizes p53, but its gene is transcriptionally activated by p53, constituting an important auto-regulatory loop^34^. We investigated the effect of PEPD on MDM2 using UM-UC-3 cells. PEPD knockdown, which activates p53 as described before, increased MDM2 expression as expected (Fig. 7a). Although PEPD siRNA alone did not alter total p53 level, combination with MG132, a specific proteasome inhibitor, elevated p53 level (Fig. 7b), indicating MDM2-mediated p53 degradation upon PEPD knockdown. In cells treated only by PEPD siRNA, it seems likely that increase in MDM2-mediated degradation of nuclear and cytosolic p53 is offset by p53 translocation to mitochondria.

We next analyzed mitochondria-free lysates prepared from UM-UC-3 cells treated with control or PEPD siRNA. As expected, PEPD siRNA treatment significantly reduced both PEPD and p53 levels but increased MDM2 level (Fig. 7c). However, despite a marked decrease in p53 level in the above-described samples, more p53 molecules were associated with MDM2 in such samples than in control samples (Fig. 7c). Conversely, while p53 co-immunoprecipitated with PEPD in the lysates, no MDM2 could be detected in the precipitates (Fig. 7d). These results show that PEPD and MDM2 compete for p53 binding. Notably, MDM2 binds to N-terminal residues 18–26 of p53^34^, not far from the PRD to which PEPD binds, which may explain why PEPD and MDM2 are mutually exclusive in binding to p53. Because MDM2 promotes p53 mitochondrial translocation by catalyzing p53 monoubiquitination^35^, we investigated whether MDM2 is involved in the translocation of nuclear and cytosolic p53 to mitochondria in response to PEPD knockdown, using UM-UC-3 cells. Cells were treated with control or MDM2 siRNA for 24 h, and then treated with control and PEPD siRNA for 48 h. Levels of MDM2 and/or PEPD were strongly reduced by the corresponding siRNA (Fig. 7e). Total p53 level increased following MDM2 knockdown, as expected, and increased further following knockdown of both MDM2 and PEPD (Fig. 7e), presumably due to increased nuclear p53 phosphorylation in response to PEPD knockdown (Fig. 4d), as phosphorylated p53 is stabilized. However, while PEPD knockdown caused p53 translocation to mitochondria as expected, such movement of p53 did not occur in cells with knockdown of MDM2 or knockdown of both MDM2 and PEPD (Fig. 7e; compare p53 level in mitochondria-free cell lysates with that in mitochondria). Nearly identical results were obtained using a different MDM2 siRNA (Supplementary Fig. 9a). Moreover, p53^mPRD^ whose mitochondrial translocation is not altered by PEPD knockdown (Fig. 6b) has little affinity to MDM2 (Supplementary Fig. 9b). These results show that PEPD-knockdown-induced mitochondrial translocation of p53 requires MDM2. MDM2-catalyzed monoubiquitination of p53 is required for nuclear exit and mitochondrial translocation of p53^35,36^. Indeed, PEPD knockdown not only increased p53 binding to MDM2 as described above but also caused marked increase in mitochondrial level of monoubiquitinated p53 under inhibition of deubiquitinylation by ubiquitin aldehyde (UbAl) (Fig. 7f). The multiple bands of monoubiquitinated p53 shown in Fig. 7f are consistent with ubiquitination of multiple lysine residues in p53 by MDM2^37^.

### Stress signals free p53 from PEPD to fully activate p53

**Fig. 8:**
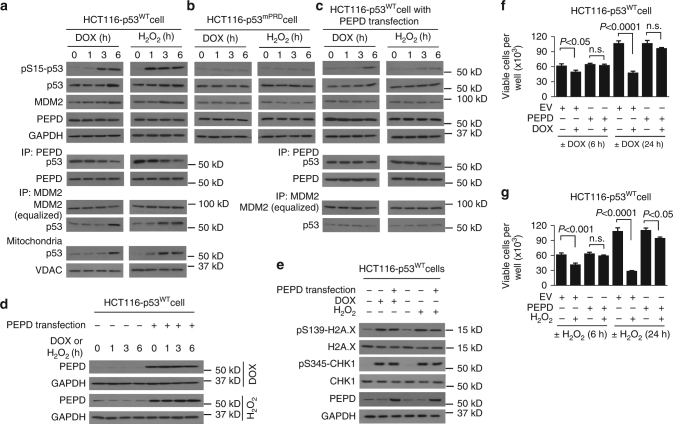
p53 activation by stress relies on p53 separation from PEPD. **a** IB and IP-IB analysis of various proteins in cell lysates and mitochondria after cell treatment with DOX or H_2_O_2_. **b** IB analysis of various proteins in cells treated with DOX or H_2_O_2_. **c** IB and IP-IB analysis of various proteins in cells transfected with PEPD for 24 h and then treated with DOX or H_2_O_2_. **d** IB analysis of PEPD in cell samples from **a** and **c**. **e** IB analysis of H2A.X, CHK1 and PEPD in control cells, cells treated with DOX or H_2_O_2_ for 6 h, or cells transfected with PEPD for 24 h and then treated with DOX or H_2_O_2_ for 6 h. **f**, **g** Measurement of cell viability. Cells were cultured in 12-well plates (2 × 10^4^ cells/well) for 24 h, transfected with EV or PEPD, and 24 h later treated with DOX, H_2_O_2_ or solvent for 6 h or 24 h. Data are means ± s.d. (*n* = 3); two-way ANOVA followed by Tukey multiple comparisons test; n.s.; not significant. GAPDH and VDAC were measured as loading controls. DOX and H_2_O_2_ were used in all experiments at 400 nM and 400 μM, respectively

More than 50% of p53 are bound to and sequestered by PEPD in cells (Fig. 5f). We wondered whether PEPD amass p53 for rapid response to stress. Both DOX (a DNA-intercalating antitumor agent) and H_2_O_2_ (an oxidative stressor) are well known to activate p53. Both agents rapidly caused p53 phosphorylation, p53 stabilization and induction of MDM2 (used as a representative p53 target protein) in HCT116-p53^WT^ cells, with H_2_O_2_ acting faster than DOX, which was accompanied by p53 disassociation from PEPD, without altering PEPD expression (Fig. 8a). We also evaluated H_2_O_2_ in UM-UC-3 cells and obtained very similar results (Supplementary Fig. 10a). However, neither agent had any activity in HCT116-p53^mPRD^ cells (Fig. 8b). Focusing on HCT116-p53^WT^ cells, we found that DOX and H_2_O_2_ also induced mitochondria translocation of p53, which is consistent with increased MDM2-p53 association (Fig. 8a). We next asked whether blocking PEPD-p53 disassociation might prevent DOX and H_2_O_2_ from activating p53. We overexpressed PEPD in cells by gene transfection (24 h) and then treated the cells with DOX or H_2_O_2_. PEPD overexpression strongly inhibited p53 disassociation from PEPD in cells exposed to DOX or H_2_O_2_ (Fig. 8c, d). In these cells, the agents were also largely unable to cause p53 phosphorylation, p53 stabilization, MDM2 induction, and MDM2 association with p53 (Fig. 8c). The slight increase in p53 phosphorylation and stability in PEPD-overexpressed cells after treatment with DOX or H_2_O_2_ likely is due to incomplete inhibition of p53 disassociation from PEPD by overexpressed PEPD. We did not examine mitochondria translocation of p53 in the PEPD-overexpressed cells, since neither agent increased MDM2 association with p53. It is important to note that PEPD overexpression did not attenuate DNA damage caused by DOX or H_2_O_2_, as measured by phosphorylation of H2A.X and CHK1 (Fig. 8e), markers of DNA damage^38^. Based on these results, we predicted that PEPD overexpression would also prevent cell death following treatment with DOX or H_2_O_2_. We carried out the same gene transfection as described above to achieve PEPD overexpression, and 24 h later, these cells along with EV-transfected cells were treated with DOX, H_2_O_2_ or solvent for 6 h or 24 h. After 6 h treatment with DOX and H_2_O_2_, 20 and 33% of EV-transfected cells died, respectively, but cell death was minimal and statistically insignificant (*P* > 0.05, ANOVA) in PEPD-transfected cells (Fig. 8f, g). After 24 h treatment with DOX and H_2_O_2_, 56 and 74% of EV-transfected cells died, but cell death in PEPD-transfected cells was only 6% (statistically insignificant; *P* > 0.05, ANOVA) for DOX and 15% for H_2_O_2_ (Fig. 8f, g). These results show that p53 dissociation from PEPD is indispensable to p53 response to the stress signals.

### Quenching ROS prevents stress-induced p53 liberation from PEPD

**Fig. 9:**
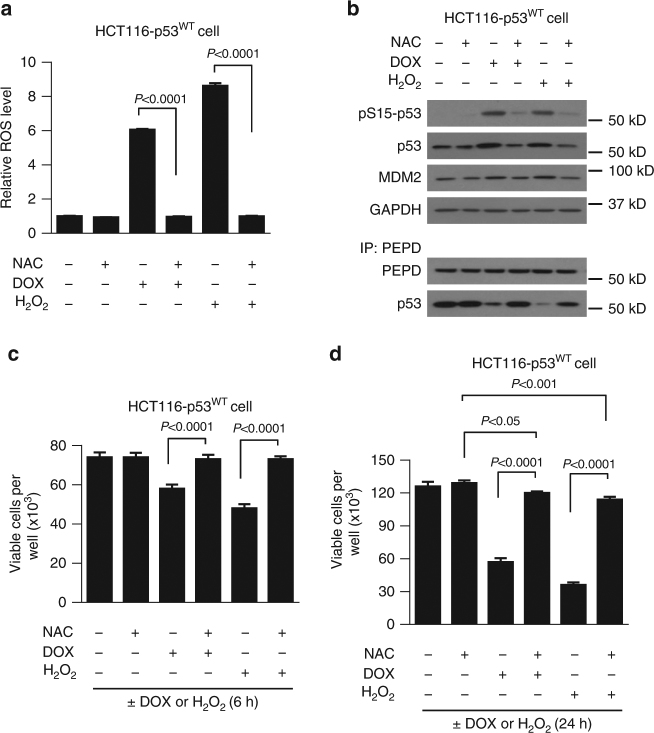
ROS is key to stress-induced p53 liberation from PEPD. **a** Relative ROS level in cells treated with solvent, DOX or H_2_O_2_ for 6 h, with or without NAC pretreatment for 3 h. **b** IB and IP-IB analysis of various proteins in cells treated with solvent, DOX or H_2_O_2_ for 6 h, with or without NAC pretreatment for 3 h. GAPDH was measured as a loading control. **c**, **d** Measurement of cell viability after treatment with DOX or H_2_O_2_ for 6 h or 24 h, with or without NAC pretreatment for 3 h. Data are means ± s.d. (*n*–3); two-way ANOVA followed by Tukey multiple comparisons test. In all experiments, cells were cultured in 12-well plates (4 × 10^4^ cells/well) for 24 h before experimental treatment. DOX, H_2_O_2_ and NAC were used at 400 nM, 400 μM and 5 mM, respectively

Both DOX and H_2_O_2_ generate reactive oxygen species (ROS) in cells, which is critical for p53 activation^39,40^. Treatment of HCT116-p53^WT^ cells with DOX (400 nM) or H_2_O_2_ (400 μM) for 6 h caused marked increase in cellular ROS, but the ROS was totally quenched, if the cells were pretreated with N-acetylcysteine (NAC) (5 mM) for 3 h (Fig. 9a). NAC is a well-known ROS scavenger. Such NAC pretreatment also blocked p53 separation from PEPD, abolished p53 activation and stabilization, inhibited MDM2 induction, and prevented cell death, when the cells were subsequently treated with DOX or H_2_O_2_, while NAC alone had no effect (Fig. 9b–d). Similar results were obtained by replacing NAC with Tempol, another ROS quencher (Supplementary Fig. 10b–e). Thus, ROS generated by the stress signals frees p53 from PEPD, to enable p53 activation.

## Discussion

We have demonstrated that under normal conditions PEPD is essential for cell survival by suppressing p53. We show that the C-terminal sequence of PEPD binds to the PRD in p53 (Fig. 5), which allows PEPD to accomplish two important tasks: (1) to prevent nuclear p53 phosphorylation in its transactivation domain (Fig. 4) and to reduce free nuclear p53 level (Fig. 5), leading to inhibition of p53 trans-activation and trans-suppression activities (Figs. 1, 2 and 4), and (2) to prevent mitochondrial translocation of nuclear and cytosolic p53 by preventing p53 from binding to MDM2 (Figs. 3, 6 and 7). PEPD sequesters >50% of cellular p53 under normal conditions (Fig. 5). PEPD modulates p53 without requiring its enzymatic activity (Fig. 1). PEPD is widely believed to exist only in the cytoplasm^2,3^, but we show that it is in both cytoplasm and nucleus and suppresses p53 in both places (Fig. 3). We also show why cells set up the PEPD-p53 complex in the first place. We show that stress signals, using H_2_O_2_ and DOX as examples, must free p53 from PEPD, via ROS, in order to achieve robust p53 activation and that the p53-PEPD complex is designed to rapidly mobilize a large amount of pre-synthesized p53 to counter stress (Figs. 8 and 9). The PEPD-p53 interaction likely operates in most if not all cells, since both proteins are expressed ubiquitously. PEPD knockout in mice may be important for further assessing the impact of PEPD on p53 in vivo, although such mice are likely embryonically lethal and PEPD enzymatic deficiency may complicate data interpretation.

**Fig. 10:**
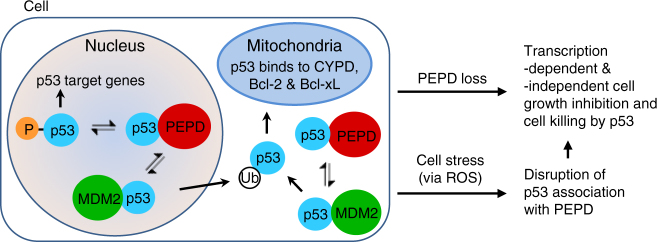
Paradigm of p53 regulation by PEPD. PEPD binds to both nuclear and cytosolic p53. More than 50% of cellular p53 is sequestered by PEPD, rendering cells dependent on PEPD for survival and growth under normal circumstances, as eliminating PEPD activates p53, which leads to cell death. PEPD binds to nuclear p53 to inhibit its phosphorylation and transcription activity. PEPD competes with MDM2 for p53 binding, thereby inhibiting MDM2-dependent mitochondria translocation of nuclear and cytoplasmic p53, leading to inhibition of transcription-independent activity of p53. The PEPD-p53 complex is designed for rapid mobilization of pre-synthesized p53 in response to stress, which is mediated by ROS. Notably, MDM2 is known to promote p53 translocation to mitochondria by monoubiquitinating p53, to promote p53 degradation by polyubiquitinating p53, and to inhibit p53 transcription activity unrelated to ubiquitination. It is also known that the MDM2 gene is transcriptionally activated by p53

MDM2 is also essential for cell survival by antagonizing p53. As mentioned before, MDM2 promotes p53 degradation by ubiquitinating it and also inhibits p53 transcription activity unrelated to ubiquitination, but MDM2 is transcriptionally activated by p53, constituting an important auto-regulatory loop. In contrast, PEPD functions as a different essential regulator of p53; it stores p53 for rapid and robust response to stress, and releasing p53 from PEPD results in cell growth inhibition or killing by p53. Moreover, PEPD competes with MDM2 for p53 binding (Fig. 7c, d) and PEPD is not regulated by p53 (Supplementary Fig. 5a). Therefore, it seems that MDM2 regulation of p53 is not only controlled by p53 reciprocally but also modulated by PEPD. A graphic presentation of p53 regulation by PEPD is shown in Fig. 10. Our results reveal a major physiological function of PEPD and also revise the paradigm of p53 regulation and p53 response to stress. Notably, only about 6% of PEPD are bound to p53 in cells normally (Fig. 5d). Since p53 level is usually kept very low in cells, there likely is a mechanism that controls p53 binding to PEPD.

Disrupting PEPD suppression of p53 may be an important therapeutic strategy in cancer. We show that PEPD knockdown in tumors in mice causes p53 activation and tumor regression. Little is known about *PEPD* gene regulation and PEPD protein degradation in cells. Agents that down regulate PEPD may be promising cancer therapeutic agents. Notably, agents that disrupt PRD-mediated protein-protein interaction have been reported^41,42^. Our data also raise the question of whether tumor cells may overexpress PEPD to enhance p53 inhibition. Indeed, a previous study found that PEPD protein expression is approximately 2-fold higher in breast cancer tissues than in normal breast tissues from untreated patients^43^.

Our study also reveals a previously unrecognized anticancer mechanism of DOX. It is currently widely accepted that DOX-induced DNA damage causes activation of certain protein kinases which in turn activate and stabilize p53. However, we show that the key step in DOX-induced p53 activation is the disruption of p53 association with PEPD via ROS. This is also true for p53 activation and cell killing by H_2_O_2_. Our finding raises the intriguing question of whether other stress-inducing anticancer agents also disrupt the PEPD-p53 complex for p53 activation. Whether antioxidant supplements should be used or avoided during chemotherapy has long been debated^44,45^. We show that the cell-killing activity of DOX is markedly reduced by NAC which scavenges DOX-generated ROS and blocks p53 separation from PEPD, thereby blocking p53 activation (Fig. 9). Our results show a significant undesirable effect of an antioxidant and provide a new research direction for further investigation of the impact of antioxidants on chemotherapy in cancer patients.

p53^mPRD^ does not bind to PEPD and is inactive in our study. Previous studies show that deletion of the entire PRD in p53 also abrogates its proapoptotic activity, but its transcriptional activity is only partially impaired^46,47^. It remains a question as to whether p53^mPRD^ may display some activity under certain conditions. Besides PEPD, several other proteins also bind and modulate p53 via PRD, such as corepressor protein mSin3a that protects p53 from proteasome-mediated degradation and is critical for p53 to repress gene expression^48,49^, transcriptional coactivator p300 that modulates DNA-dependent acetylation of p53^50^, and prolyl isomerase Pin1, important for p53 activation^51,52^. PEPD may compete with the aforementioned proteins for binding to p53. Indeed, we show that PEPD and MDM2, the latter of which binds to p53 in a sequence (aa 18–26) adjacent to PRD (aa 64–89), compete for p53 binding. p53 deleted of PRD was shown to have enhanced affinity for MDM2 and enhanced MDM2-mediated degradation^53^, likely due to the inability of the p53 deletion mutant to bind to PEPD, in light of our findings. However, the expression levels of p53^mPRD^ and p53^WT^ are similar in cells either stably or transiently expressing these proteins in our study. Notably, p53^mPRD^ has almost no binding affinity towards MDM2 (Supplementary Fig. 9b), but p53 can be degraded by MDM2-independent pathways^54,55^.

Finally, PD has been long believed to be caused exclusively by loss of PEPD enzymatic activity. Our results suggest that p53 may be hyperactive in PD cells. Indeed, dermal fibroblasts from PD patients die in culture, showing cytosolic vacuolization, mitochondrial swelling and MMP loss^56^, which seems to fit with p53 hyperactivity. Notably, two enzymatically deficient PEPD mutants that are linked to PD, including PEPD^G278D^ and PEPD^G448R^ ^17,57^, can bind to p53 (Fig. 5b), and introducing PEPD^G278D^ to PEPD-depleted cells attenuates cell death (Fig. 1b). However, the expression of PEPD^G448R^ and other PEPD mutants in fibroblasts of PD patients was found to be markedly reduced, presumably due to perturbed folding and instability of the mutant proteins^58^. Comparison of p53 activity between PD cells and control cells and evaluation of the protective activity of a p53 inhibitor, such as pifithrin-α, may clarify the role of p53 in PD and may also identify a new therapeutic strategy for PD.

In conclusion, PEPD sequester > 50% cellular p53. Disrupting PEPD association with p53 frees p53 and unleashes its transcription-dependent and -independent functions, causing cell death and tumor regression. The PEPD-p53 complex is set up for robust p53 response to stress, and p53 separation from PEPD is a prerequisite for robust p53 activation by stress signals.

## Methods

### Antibodies

Catalog numbers and dilution fold are listed in parentheses. The following antibodies were purchased from Cell Signaling Technology: Anti-Bak (3792; 1:1000), anti-Bax (2772; 1:1000), anti-Bcl-2 (2870; 1:1000), anti-Bcl-xL (2764; 1:500), anti-CHK1 (2360; 1:1000), anti-phospho-CHK1 (S345; 2348; 1:1000), anti-cleaved caspase-3 (9661; 1:1000), anti-cleaved caspase-7 (9491; 1:1000), anti-cleaved caspase-8 (9496; 1:500), anti-cleaved caspase-9 (9501; 1:1000), anti-cytochrome c (4272; 1:500), anti-H2A.X (7631; 1:1000), anti-phospho-H2A.X (S139; 9718; 1:1000), anti-p53 (2524; 1:1000), anti-p53 (2527; 1:1000), anti-phospho-p53 (S6; 9285; 1:1000), anti-phospho-p53 (S15; 9284; 1:1000), anti-p21 (2946; 1:1000), and anti-voltage-dependent anion channel (VDAC; 4866; 1:1000). The following antibodies were purchased from Santa Cruz Biotechnology: Anti-CD95 (1023; 1:500), anti-cyclophilin 40, also known as anti-CYPD (137157; 1:400), anti-His tag (803; 1:500), anti-lamin A (20680; 1:1000), anti-MDM2 (965; 1:500), and anti-ubiquitin (8017; 1:200). Anti-PEPD (Ab86507; 1:500) and anti-glyceraldehyde-3-phosphate dehydrogenase (GAPDH; MAB374; 1:5000) were purchased from Abcam and EMD Millipore, respectively. Anti-mouse IgG-horseradish peroxidase (IgG-HRP; NA931V; 1:4000–5000) and anti-rabbit IgG-HRP (NA934V; 1:5000) were purchased from GE Healthcare.

### Chemicals, biochemicals, and enzymes

Catalog numbers are listed in parentheses. The following reagents were purchased from Sigma-Aldrich: β-mercaptoethanol (M6250), dithiothreitol (D0632), 3-(4,5-Dimethylthiazol-2-yl)-2,5-diphenyltetrazolium bromide (MTT; M2128), dimethyl sulfoxide (DMSO; M81802), doxorubicin (44583), Gly-Pro (G3002), MG132 (M7479), N-acetyl-cysteine (A7250), phenylmethanesulfonyl fluoride (PMSF; 329-98-6), phosphatase inhibitor cocktail 2 (P5726), proline (P0380), propidium iodide (PI; P-4170), puromycin (P8833), rhodamine 123 (62669-70-9), Tempol (581500) and trypan blue (T8154). Blasticidin (R21001), CM-H_2_DCFDA (C6827), MluI restriction enzyme (ER0561), RNase (AM2286) and SgfI restriction enzyme (FD2094) were purchased from Thermo Fisher Scientific. A protease inhibitor cocktail (11-836-153-001) was purchased from Roche Applied Science. G-sepharose beads (17-6002-35) was purchased from GE Healthcare. Hydrogen peroxide 3% USP (F0010) was purchased from Hydrox Laboratories. Matrigel (356237) was purchased from Corning. Phos-tag acrylamide (304-93562) was purchased from Wako. Pifithrin-α (1267) was purchased from R&D Systems. Sodium dodecyl sulfate (SDS; 161-0301) was purchased from Bio-Rad. Ubiquitin aldehyde (UbAl; BML-UW8450) and Z-VAD-FMK (ALX-260-020-M001) were purchased from Enzo Life Sciences.

### Commercial assay kits

Catalog numbers are listed in parentheses. Bicinchoninic acid assay kit (BCA; reagent A: 23228; reagent B: 1859078) was purchased from Pierce. Cell lysis buffer (9803) was purchased from Cell Signaling. FuGENE HD (E231A) was purchased from Promega. Lipofectamine RNAiMAX (13778-075) was purchased from Invitrogen. NE-PER nuclear and cytoplasmic extraction kit (78833) was purchased from Thermo Fisher Scientific. Nickel nitrilotriacetic acid-agarose chromatography (30210) was purchased from Qiagen. QuikChange Lightning Site-Directed Mutagenesis kit (210518) and QuikChange Lightning Multi Site-Directed Mutagenesis kit (210515) were purchased from Agilent Technologies. Silver staining kit (24612) was purchased from Thermo Fisher Scientific. Luminata Classico (WBLUC0500) and Luminata Cresendo (WBLUR0100) were purchased from Millipore.

### Commercial plasmids

Catalog numbers are listed in parentheses. Following plasmids were purchased from Addgene: PG13-luc (16442), MG15-luc (16443), pET15b-His-human p53 (24859), pET15b-His-human p53^1-320aa^ (24864), pET15b-His-human p53^94-312aa^ (24866), pET15b-His-humanp53^82-393aa^ (24867). pCMV-XL5-human PEPD (SC119982) was purchased from Origene. pRL-TK (E2241) was purchased from Promega.

### Plasmids made previously in our lab

pBAD/TOPO-human PEPD-His and pBAD/TOPO-human PEPD^G278D^-His were reported previously^8^. pBAD/TOPO-human PEPD^1-184aa^-His, pBAD/TOPO-human PEPD^1-265aa^-His and pBAD/TOPO-human PEPD^265-493aa^-His were also reported previously^9^.

### Construction of new plasmids

Bacterial expression plasmid pET15b-His-human p53 (Addgene) was used as a template to generate mutation of proline to alanine as well as specific deletion in p53 coding region, including deletion of 81–94 aa (p53^del81-94aa^) and mutation of 11 prolines to alanines in 64-89 aa region (p53^mPRD^), using QuikChange Lightning Site-Directed Mutagenesis Kit or QuikChange Lightning Multi Site-Directed Mutagenesis Kit. The sequences of the primers used for the reactions are provided below. For mammalian expression of human p53 and p53^mPRD^, pET15b-His-human p53 and pET15b-His-human p53^mPRD^ were used as templates to amplify p53 or p53^mPRD^, respectively using 5′-GCGATCGCatggaggagccgcagtcaga-3′ as the SgfI-forward primer and 5′-ACGCGT tcagtctgagtcaggcccttct-3′ as the MIuI-reverse primer. The amplified PCR products were digested by SgfI and MIuI and subcloned into pCMV6-A-Puro to generate pCMV6-A-human p53-puro and pCMV6-A-human p53^mPRD^-puro. The plasmid expressing PEPD^G448A^ (pBAD/TOPO-PEPD^G448A^-His) was generated using the pBAD/TOPO-PEPD-His^8^ as a template and the QuikChange Lightning Site-Directed Mutagenesis Kit, using the primers provided below. All primers were purchased from IDT. All constructs were confirmed by DNA sequence analysis.Primers for generating pET15b-His-human p53^del81-94aa^: 5′-ctgcaccagcagctccttcttctgtcccttc-3′ (forward); 5′-gaagggacagaagaaggagctgctggtgcag-3′ (reverse). Primers for generating PEG15b-His-human p53^mPRD^: 5′-ccagatgaagctgccagaatggcagaggctgctg-3′ (forward 1); 5′-ctgctgccgccgtggccgctgcagcagcagctgctacag-3′ (forward 2); 5′-acagcggcggccgctgcagcagccgcctcctggccc-3′ (forward 3). Primers for generating pBAD/TOPO-PEPD^G448A^-His: 5′-cgcggttttggcagggtccgcatcg-3′ (forward); 5′-cgatgcggaccctgccaaaaccgcg-3′ (reverse).

### Preparation of recombinant proteins

Recombinant human p53 and its mutants, as well as recombinant human PEPD and its mutants were expressed in *E.coli*. p53 and its mutants are N-terminal His tagged, whereas PEPD and its mutants are C-terminal His tagged. The bacterial expression constructs include pET15b-His-human p53, pET15b-His-human p53^1-320aa^, pET15b-His-human p53^94-312aa^, pET15b-His-human p53^82-393aa^, pET15b-His-human p53^del81-94aa^, pET15b-His-human p53^mPRD^, pBAD/TOPO-human PEPD-His, pBAD/TOPO-human PEPD^G278D^-His, pBAD/TOPO-human PEPD^G448A^-His, pBAD/TOPO-human PEPD^1-184aa^-His, pBAD/TOPO-human PEPD^1-265aa^-His, and pBAD/TOPO-human PEPD^265-493aa^. Each protein or peptide was purified by nickel nitrilotriacetic acid-agarose chromatography and concentrated in PBS using Ultracel YM-10 or YM-30 Centricons (Millipore; catalog numbers: MRCPRT010 and MRCF0R030, respectively). The purity of each protein and peptide was confirmed by SDS–PAGE and silver staining, using a silver staining kit (see Supplementary Fig. 7b and d).

### Cell and cell culture

UM-UC-3 cells (ATCC; catalog number: CRL-1749) were cultured in McCoy’s 5 A supplemented with 10% fetal bovine serum (FBS). HCT116 cells (ATCC; catalog number: CCL-247) and their sublines were cultured in high-glucose DMEM supplemented with 10% FBS. Normal human urothelial cells (HUC) isolated from human bladder, which were purchased from ScienCell Research Laboratories (catalog number: 4320), were cultured in urothelial cells medium (ScienCell Research Laboratories; catalog number: 4321). UROtsa cells were generated from a primary culture of normal human urothelium through immortalization with a construct containing the SV40 large T antigen^59^ and were generously provided by Dr. Scott H. Garrett at University of South Dakota. RUOtsa cells were cultured in high-glucose DMEM supplemented with 10% FBS. 32D cells were generously provided by Dr. Gibbes R. Johnson of US Food and Drug Administration and were cultured in RPMI-1640 medium supplemented with 10% FBS, 5%WEHI-3B cells-conditioned medium and 0.1% β-mecaptoethanol^8^. All cell lines were mycoplasma-free. UM-UC-3 cells and HCT116 cells including the sublines were authenticated using short tandem repeat (STR), which was carried out at the Genomic Shared Resources, Roswell Park Cancer Institute. All cells were cultured in humidified incubators at 37 ^o^C with 5% CO_2_. Cell culture media were purchased from Corning Cellgro, including McCoy’s 5 A (catalog number: 10-050-CV), RPMI-1640 (catalog number: 10-040-CV), and high glucose DMEM (catalog number: 10-013-CV). FBS was purchased from Gibco (catalog number: 10437).

### PEPD knockout by CRISPR/Cas9

Human PEPD genedit CRISPR Cas9 nuclease & gRNA target gene knockout set was purchased from Celltechgen (CTG-CS9O-19761), including the CRISPR Cas9 nuclease expression vector (pST1374-N-NLS-Flag-Cas9-EGFP), PEPD gRNA vector 1 (pGL3-PEPD-sgRNA1), PEPD gRNA vector 2 (pGL3-PEPD-sgRNA2) and the scramble RNA vector. The human PEPD-specific gRNA sequences are as follows: gccgctcacacaggcgctgc (gRNA1) and gcggaagaaccctgctgtgc (gRNA2). Cells were grown in 6-well plates with 2 ml medium per well (UM-UC-3: 1.2 × 10^5^  cells/well; HUC: 1.5 × 10^5^ cells/well; UROtsa: 1.2 × 10^5^ cells/well) and 24 h later were transfected with 2 μg of each specific plasmid per well with combination of Cas9 plus scramble RNA, Cas9 plus PEPD gRNA1, and Cas9 plus PEPD gRNA2, respectively, using FuGENE HD for 48 h, followed by selection with puromycin (1–2 μg/ml) and blasticidin (7.5–10 μg/ml). The specific concentrations of the antibiotics were selected based on their cell kill curves. The cells were examined at 48 h after plasmid transfection and also after 72 h treatment with the antibiotics microscopically. UM-UC-3 cells were examined using Axiovert 40 CFL (Carl Zeiss). Images were taken using A-plan ×5 or ×10 objective lenses (Carl Zeiss) and a Flex Camera (Spot) using the Spot advanced acquisition software. HUC and UROtsa cells were examined uing IX73 (Olympus). Images were taken using UPlanFL ×4 or ×10 objective lenses (Olympus) and a DP80 Camera (Olympus) using the cellSens standard software.

### Gene or siRNA transfection and other treatments

Transfection of pCMV6-XL5 plasmid (empty vector), pCMV6-XL5-PEPD (expressing human PEPD), pCMV6-A-human p53-puro (expressing p53^WT^) and pCMV6-A-human p53^mPRD^-puro (expressing p53^mPRD^) was performed using FuGENE HD. Cells were grown either in 12-well plates (2 × 10^4^ cells/well with 1 ml medium) or 6-well plates (1 × 10^5^ cells/well with 2 ml medium) for 24 h and then transfected with 1 or 2 μg DNA per well for 24 h. In experiments where plasmid transfection was followed by treatment with DOX or H_2_O_2_, cells were first transfected with the plasmid and 24 h later treated with each agent or solvent control for up to 24 h. In experiments where gene transfection follows siRNA transfection, cells were first transfected with siRNA as described below and 48 h later transfected with the plasmid for 48 h. For transfection of plasmids including PG13-Luc, MG15-luc and pRL-TK, cells were grown in 6-well plates (2 × 10^5^ cells/well with 2 ml medium) for 24 h and then transfected with 2 μg DNA per well for 24 h.

For siRNA transfection, cells were grown in 6-well plates (5–10 × 10^4^ cells/well with 2 ml medium), 12-well plates (2 × 10^4^ cells/well with 1 ml medium) or 96-well plates (2 × 10^3^ cells/well with 0.15 ml medium) for 24 h and then transfected with control siRNA, PEPD siRNA and/or MDM2 siRNA (10 nM each) using Lipofectamine RNAiMAX for up to 72 h. In cells transfected with PG13-Luc, MG15-Luc and pRL-TK, siRNA transfection was performed 24 h after plasmid transfection. In experiments where cells were treated with siRNA and also treated with PEPD or PEPD^G278D^, PEPD or PEPD^G278D^ were added to the culture medium 24 h after siRNA transfection. In experiments where cells were transfected with siRNA and also treated with pifithrin-α, pifithrin-α or solvent was added to the culture medium together with the siRNA. In experiments where cells were transfected with siRNA and also treated with UbAl, UbAl or solvent was added to the cultured medium during the final 4 h of culture. All siRNAs were purchased from Origene, including nonspecific scrambled control siRNA (Catalog number: SR3004; 

sequence: rCrGrU rUrArA rUrCrG rCrGrU rArUrA rArUrA rCrGrC rGrUA T), PEPD siRNA

(Catalog number: SR303443; sequence: rCrGrA rArGrU rCrArA rCrArA rUrArC rCrArU rUrCrU rUrCA C), MDM2 siRNA #1(Catalog number: SR302849A; sequence: rCrCrC rUrArG rGrArA rUrUrU rArGrA rCrArA rCrCrU rGrAA A), MDM2 siRNA #2 (Catalog number: SR302849B; sequence: rGrUrA rCrUrA rGrArC rArArC rArUrG rUrArA rUrUrA rArUG A).

In experiments where cells were treated with the following agents without gene transfection or siRNA transfection, cells were grown in 6-well plates (5 × 10^4^ cells/well with 2 ml medium) or 12-well plates (2 × 10^4^ cells/well with 1 ml medium) for 24 h and then treated with recombinant human PEPD, recombinant human PEPD^G278D^, pifithrin-α, MG132, UbAl, DOX or H_2_O_2_. In some experiments, cells were pretreated with NAC for 3 h or Tempol for 4 h, washed with PBS, and then treated in fresh medium with DOX or H_2_O_2_. Pifithrin-α and MG132 were prepared in DMSO. DOX, H_2_O_2_, NAC, Tempol and UbAl were prepared in distilled water. PEPD and PEPD^G278D^ were prepared in PBS. Final solvent concentration in culture medium was ≤0.1%.

### Generating cells stably expressing p53^WT^ or p53^mPRD^

HCT116-p53^−/−^ cells grown in 6-well plates were transfected with pCMV6-A-human p53-puro or pCMV6-A-human p53^mPRD^-puro (2 μg DNA/well) using FuGENE HD. The cells were treated with puromycin (6 μg/ml) at 72 h after gene transfection. The culture medium containing puromycin was changed twice weekly. After one week of puromycin treatment, the cells were subcultured to 96-well plates (1 cell/well) and ~4 weeks later, proliferating cells in a given well were subcultured and propagated in 24-well plates and analyzed by IB for transgene expression, to identify desired stable clones, which were further propagated in 10-cm dishes.

### Preparation of cell and tumor samples

To prepare whole cell lysates, cells were washed with PBS twice, mixed with 1× cell lysis buffer (Cell Signaling) supplemented with 2 mM PMSF and a protease inhibitor cocktail from Roche Applied Science, placed on ice for 10 min, sonicated at 0–4 ^o^C to enhance cell lysis using a Branson Model 450 sonifier, and finally centrifuged at 13,000×*g* for 10 min at 4 ^o^C, and the supernatant fraction is collected as whole cell lysate. To prepare cell lysates minus mitochondria as well as mitochondria fraction, cells were washed with ice-cold PBS, placed on ice for 10 min, suspended in isotonic homogenization buffer (25 mM sucrose, 10 mM KCl, 1.5 mM MgCl_2_, 1 mM NaEDTA, 1 mM NaEGTA, 1 mM dithiothreitol, 1 mM PMSF, 0.1 mM Tri–HCl, pH 7.4, and a protease inhibitor cocktail mentioned above) at 10 × 10^6^ cells per 0.75 ml buffer, and then homogenized in a Dounce homogenizer. The homogenates were centrifuged at 400×*g* for 5 min at 4 ^o^C. The precipitates were resuspended in 0.1 ml cell lysis buffer per sample (sample A), whereas the supernatant fraction was further centrifuged at 12,000×*g* for 20 min at 4 ^o^C, to obtain cytosolic fraction (supernatant) and mitochondria fraction (pellet). When the cytosolic fraction was combined with sample A mentioned above, the mixture is designated as cell lysates minus mitochondria. The mitochondria pellets were washed three times with the homogenization buffer and then suspended in 1× lysis buffer supplemented with 2 mM PMSF and the protease inhibitor cocktail mentioned above. Nuclear fraction was prepared using the NE-PER Nuclear and Cytoplasmic Extraction kit, following the manufacturer’s instruction. Tumor samples were mixed with RIPA buffer (25 mM Tris–HCl, PH7.6, 150 mM NaCl, 1% Nonidet P-40, 1% sodium deoxycholate, and 0.1% SDS), which was supplemented with 2 mM PMSF, the protease inhibitor cocktail mentioned above, and phosphatase inhibitor cocktail 2 from Sigma-Aldrich, and homogenized in a Dounce homogenizer. The homogenates were cleared by centrifugation at 13,000×*g* for 15 min at 4 ^o^C.

### Immunoblotting and immunoprecipitation

Protein concentrations in all samples were measured using the BCA kit. For immunoblotting (IB), samples were mixed with 4x loading dye, heated for 5 min at 95 ^o^C, and resolved by SDS-PAGE (8–12.5%). Proteins were transferred to polyvinylidene fluoride membrane, probed with specific antibodies, and detected using either Luminata Classico (Millipore) or Luminata Cresendo (Millipore). Certain IB bands were quantified by ImageJ (NIH Image). For phos-tag IB, MnCl_2_ (100 μM final concentration) and phos-tag acrylamide (20 μM final concentration) was added to the regular resolving gel. Notably, phos-tag provides phosphate affinity SDS-PAGE, generating mobility shift of phosphorylated proteins. For immunoprecipitation (IP), cell lysates (0.5 mg protein/sample) or mitochondrial samples (0.1 mg protein/sample) were incubated with a desired antibody overnight at 4 ^o^C, followed by incubation of 500 μl sample with 30 μl G-Sepharose beads (2 mg/ml) for 1 h at room temperature. The beads were washed three times with IP buffer, suspended in 2x SDS loading buffer, boiled for 5 min, and analyzed by IB. Notably, the uncropped scans of the most important IBs are shown in Supplementary Figs. 11–19.

### Measurement of direct binding between p53 and PEPD

Binding reactions were carried out in PBS in a total volume of 100 µl for 2 h at room temperature. To assess binding of PEPD to human p53 and its mutants, PEPD at 100 nM was incubated with a potential binding partner at 200 nM. At the end of the incubation, 300 µl PBS containing a p53 antibody (2 μg) which recognizes p53 and all its mutants was added to the incubation solution, which was further incubated at 4 °C overnight, followed by pull-down with protein G-agarose. To compare PEPD and its mutants for binding to p53, PEPD or a mutant at 100 nM was incubated with p53 at 200 nM. At the end of the incubation, 300 µl PBS containing a PEPD antibody (2 μg) which recognizes PEPD and all its mutants was added to the incubation solution, which was further incubated at 4 °C overnight, followed by pull-down with protein G-agarose. The immunoprecipitates were analyzed by IB.

### Measurement of cell survival

Cell survival was measured by trypan blue expulsion assay. Cells after various treatments were trypsinized and suspended in fresh culture medium. In total, 10 μl cell suspension (~550 cells) was mixed with 10 µl of 0.4% trypan blue solution, which was loaded onto a hemocytometer, and viable cells (unstained cells) were counted under an inverted microscope.

### MTT cell proliferation assay

Cells were grown in 96-well plates for 24 h and then treated with control siRNA or PEPD siRNA, as described before; 48 or 72 h later, the cells were treated with MTT at 9.2 mM for 3 h at 37 °C, washed once with PBS (0.15 ml/well) and mixed with DMSO (0.15 ml/well). Cell density in each well was determined by measuring the reduction of MTT to formazan spectroscopically at 570 nm using a microtiter plate reader.

### Cell cycle analysis

Cells were cultured in 6-well plates (5 × 10^4^ cells in 2 ml medium per well) for 24 h and then treated with control siRNA or PEPD siRNA. Following the experimental treatment, the cells were trypsinized, washed twice with ice-cold PBS, and then pelletized by centrifugation at 2,000 rpm for 5 min at 4 °C. For each sample, 1 × 10^6^ cells were suspended in 0.5 ml PI staining buffer containing 20μg/ml RNase and 50 μg/ml PI, and incubated at room temperature in the dark for 30 min. The stained cells were analyzed by a flow cytometer (BD FACS Calibur, BD Biosciences), counting 25,000 cells per sample. Cell cycle distribution was modeled using the ModFit LT software.

### Measurement of MMP

Cells were grown in 6-well plates (2 × 10^5^ cells/well with 2 ml of medium) for 24 h and then treated with siRNA, as described before; 72 h later, the cells were washed twice with ice-cold PBS, trypsinized and pelletized by centrifugation at 2,000 rpm for 5 min at 4 °C. Each cell pellet was resuspended in fresh medium at 5 × 10^5^ cells/ml and then incubated with rhodamine 123 at 10 μg/ml for 30 min at 37 °C. The cells were then washed twice with PBS, resuspended in 0.5 ml fresh medium, and measured by a flow cytometer (BD FACS Calibur, BD Biosciences), counting 10,000 cells per sample. Change in MMP was analyzed using the WinMDI 2.8 software.

### Measurement of cellular ROS

HCT116-p53^WT^ cells or UM-UC-3 cells (4 × 10^4^ per well in 12-well plate) were pretreated with or without NAC (5 mM) for 3 h or Tempol (0.5 mM) for 4 h, washed with fresh medium, and treated with either DOX (400 nM) or H_2_O_2_ (400 μM) for 6 h. The cells in each well were then harvested by trypsinization, washed twice with PBS, and suspended in 500 μl PBS. To quantify ROS level, aliquots of 100 μl cell suspension were pipetted into each well of 96-well plates and mixed with 10 μl PBS containing CM-H2DCFDA at the final concentration of 10 μM, which was incubated for 40 min at 37 °C. Fluorescence intensity (excitation at 492 nm and emission at 525 nm) was measured using a SpectraMax GeminiXS microplate reader (Molecular Devices). ROS level was normalized to the number of viable cells, which was determined by the trypan blue expulsion assay.

### Mouse tumor model and experimental treatment

We established subcutaneous tumors by inoculating UM-UC-3 cells, HCT116-p53^+/+^ cells or HCT116-p53^−/−^ cells to the flanks of male athymic nude mice (Harlan) at 8 weeks of age. One million UM-UC-3 cells were inoculated to each site in 0.1 ml of 50% Matrigel in PBS. Two million HCT116-p53^+/+^ cells or HCT116-p53^−/−^ cells were inoculated in 0.1 ml PBS to each site without Matrigel. Tumor-bearing mice were randomized cage-wise using Research Randomizer (www.randomizer.org) and when their tumors reached approximately 100 mm^3^ began treatment with intratumoral injection of either 0.1 ml PEPD siRNA (100 nM) or 0.1 ml control siRNA (100 nM). The siRNAs were prepared in Opti-MEM Reduced Serum Medium (Gibco, 31985-070). Intratumoral injection was repeated every 3–4 days for a total of 4 injections for UM-UC-3 tumors and was repeated every 2–3 days for a total of 4–5 injections for the other tumors. Tumors were measured before each injection using a caliper and their volumes were calculated using the equation of length×width^2^/2. Mice were killed at 72 h after the final treatment (UM-UC-3 tumors) or at 24 h after the final treatment (HCT116-p53^+/+^ tumors and HCT116-p53^−/−^ tumors), and tumors were removed from the mice for molecular analysis. Sample size of 7–8 per group was used, which was estimated to detect at least 50% inhibition in the intervention group with more than 85% power. The expected inhibitory efficacy of PEPD siRNA was based on data from cultured cells. The animal experiment was approved by the Institutional Animal Care and Use Committee at the Roswell Park Cancer Institute under protocol 1022 M.

### Statistical analysis

Data were analyzed by two-sided *t*-test for two-group comparison or analysis of variance (ANOVA) for multi-group comparisons (followed by Tukey multiple comparisons test), using GraphPad Prism 6 software. *P* value of 0.05 or lower was considered statistically significant.

### Data availability

The authors declare that all the data supporting the findings of this study are available within the article and its supplementary information file and from the corresponding author upon reasonable request.

## Electronic supplementary material

Supplementary

Peer Review File
