# Supplementary material for: PEPD is a pivotal regulator of p53 tumor suppressor
Source: Nat Commun. 2017 Dec 12;8:2052. doi: 10.1038/s41467-017-02097-9 (PMC5727116; doi:10.1038/s41467-017-02097-9)
Supplement: Supplementary file 1 — Supplementary [file 41467_2017_2097_MOESM1_ESM.pdf]

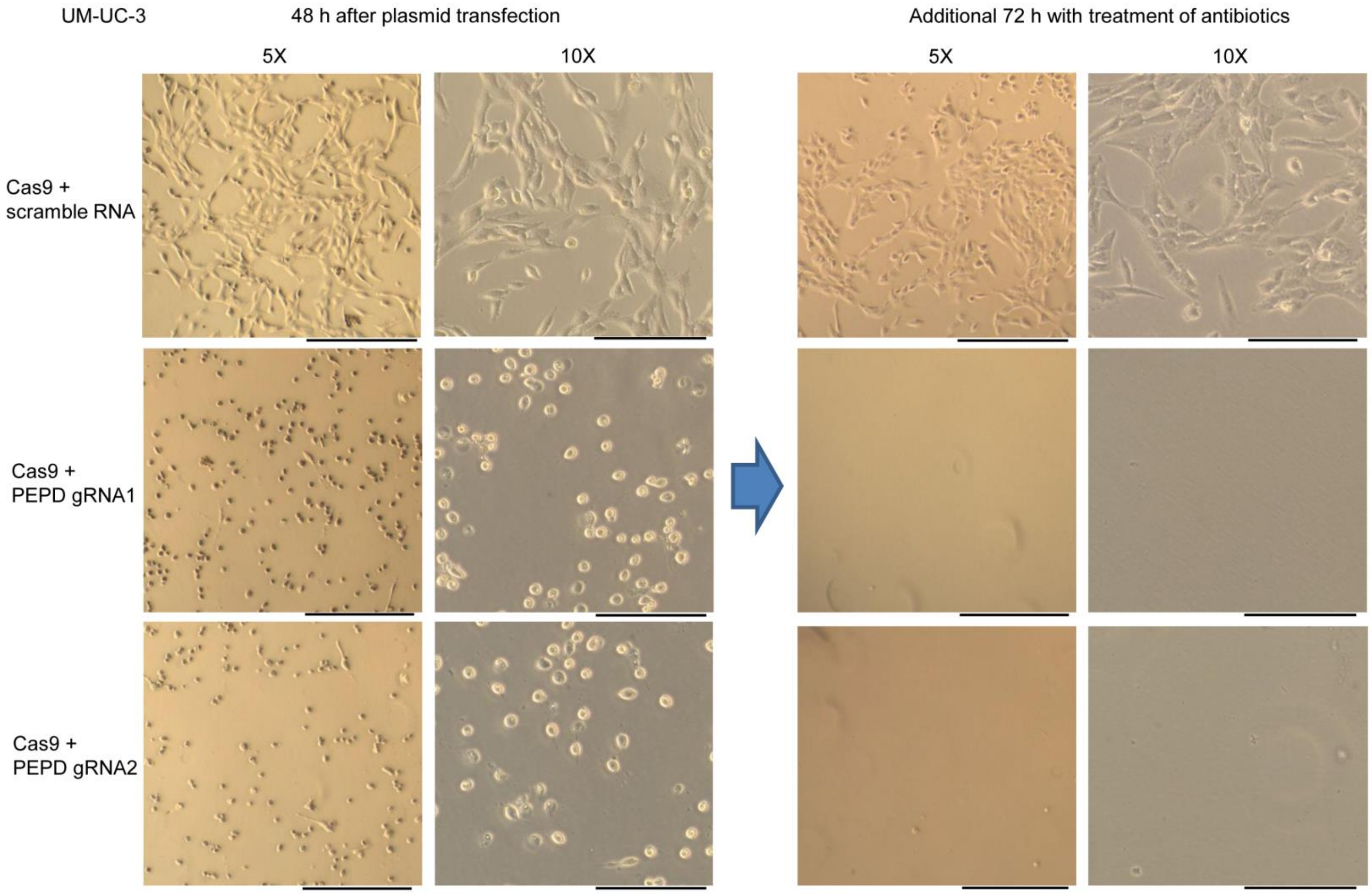

Supplementary Figure 1. PEPD knockout results in rapid and complete cell killing. UM-UC-3 cells were grown in 6-well plates and transfected with Cas9 together with scramble RNA, PEPD gRNA1 or PEPD gRNA2; 48 h later, the culture medium was replaced with fresh medium containing selection antibiotics. Representative images of cell morphology were taken (5x and 10x magnification) at 48 h following transfection and at 72 h following antibiotic treatment. These images are representative of cells in two wells treated with the same condition. Whereas cells transfected with Cas9 plus scramble RNA grew normally, those transfected with Cas9 plus PEPD gRNA1 or gRNA2 almost all rounded up at 48 h and all died after another 72 h culture with selection antibiotics, indicating that PEPD knockout is absolutely lethal. Scale bars: 400 μm (5x) and 200 μm (10x).

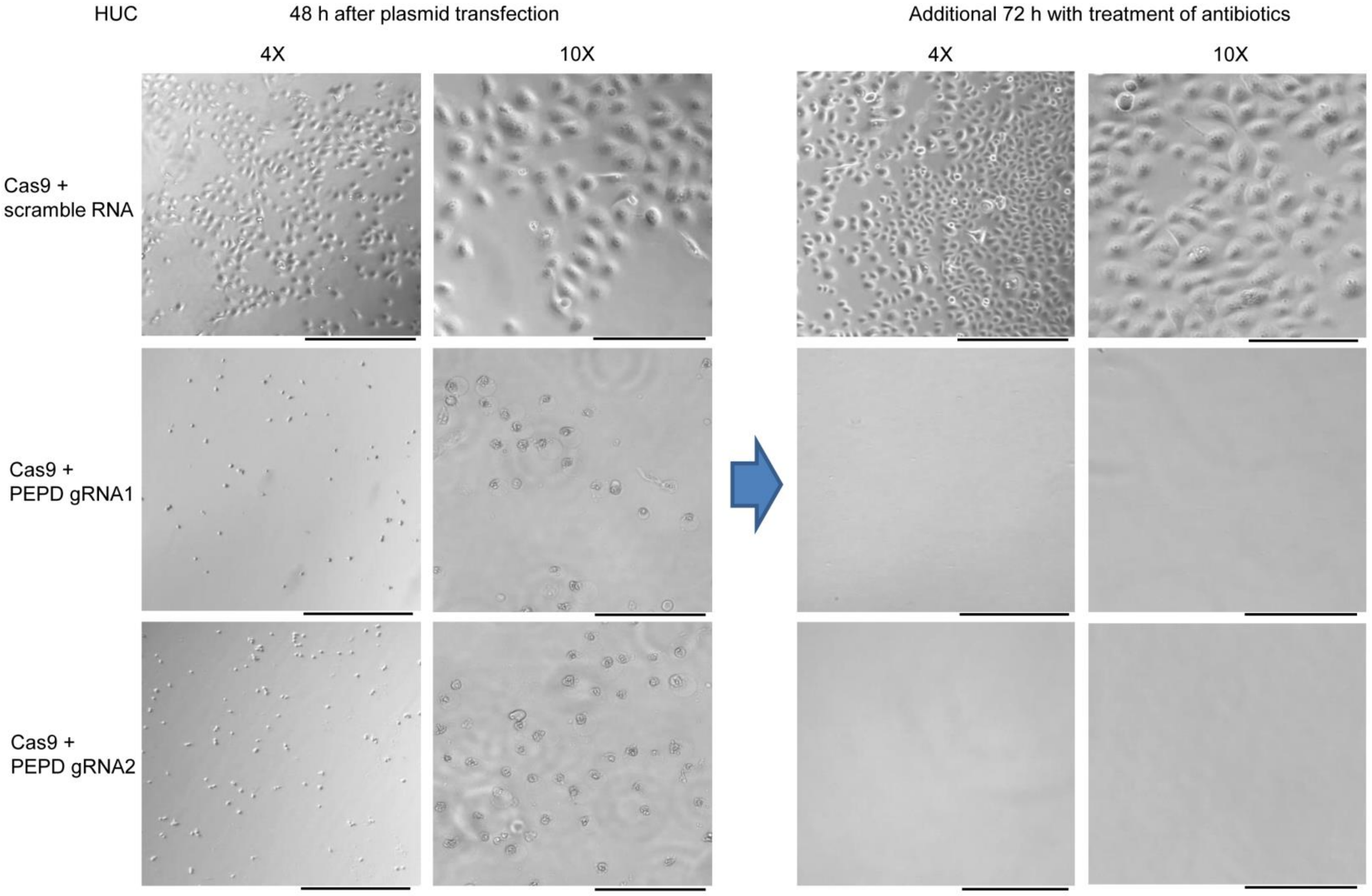

Supplementary Figure 2. PEPD knockout results in rapid and complete cell killing. Normal human urothelial cells (HUC) were grown in 6-well plates and transfected with Cas9 together with scramble RNA, PEPD gRNA1 or PEPD gRNA2; 48 h later, the culture medium was replaced with fresh medium containing selection antibiotics. Representative images of cell morphology were taken (4x and 10x magnification) at 48 h following transfection and at 72 h following antibiotic treatment. These images are representative of cells in two wells treated with the same condition. Whereas cells transfected with Cas9 plus scramble RNA grew normally, those transfected with Cas9 plus PEPD gRNA1 or gRNA2 almost all rounded up at 48 h and all died after another 72 h culture with selection antibiotics, indicating that PEPD knockout is absolutely lethal. Scale bars: 500 μm (4x) and 200 μm (10x).

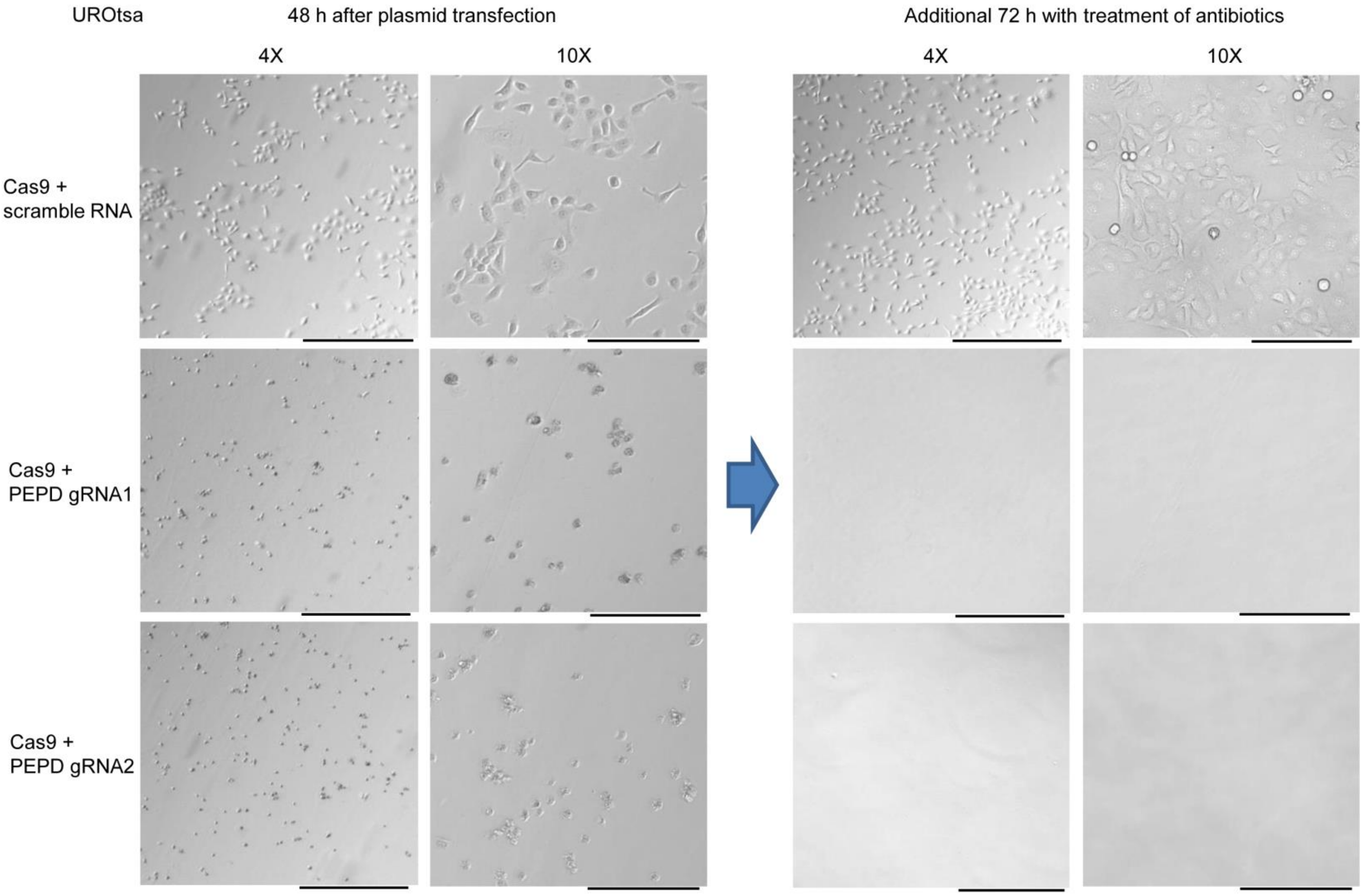

Supplementary Figure 3. PEPD knockout results in rapid and complete cell killing. Immortalized human urothelial cells (UROtsa) were grown in 6-well plates and transfected with Cas9 together with scramble RNA, PEPD gRNA1 or PEPD gRNA2; 48 h later, the culture medium was replaced with fresh medium containing selection antibiotics. Representative images of cell morphology were taken (4x and 10x magnification) at 48 h following transfection and at 72 h following antibiotic treatment. These images are representative of cells in two wells treated with the same condition. Whereas cells transfected with Cas9 plus scramble RNA grew normally, those transfected with Cas9 plus PEPD gRNA1 or gRNA2 almost all rounded up at 48 h and all died after another 72 h culture with selection antibiotics, indicating that PEPD knockout is absolutely lethal. Scale bars: 500 μm (4x) and 200 μm (10x).

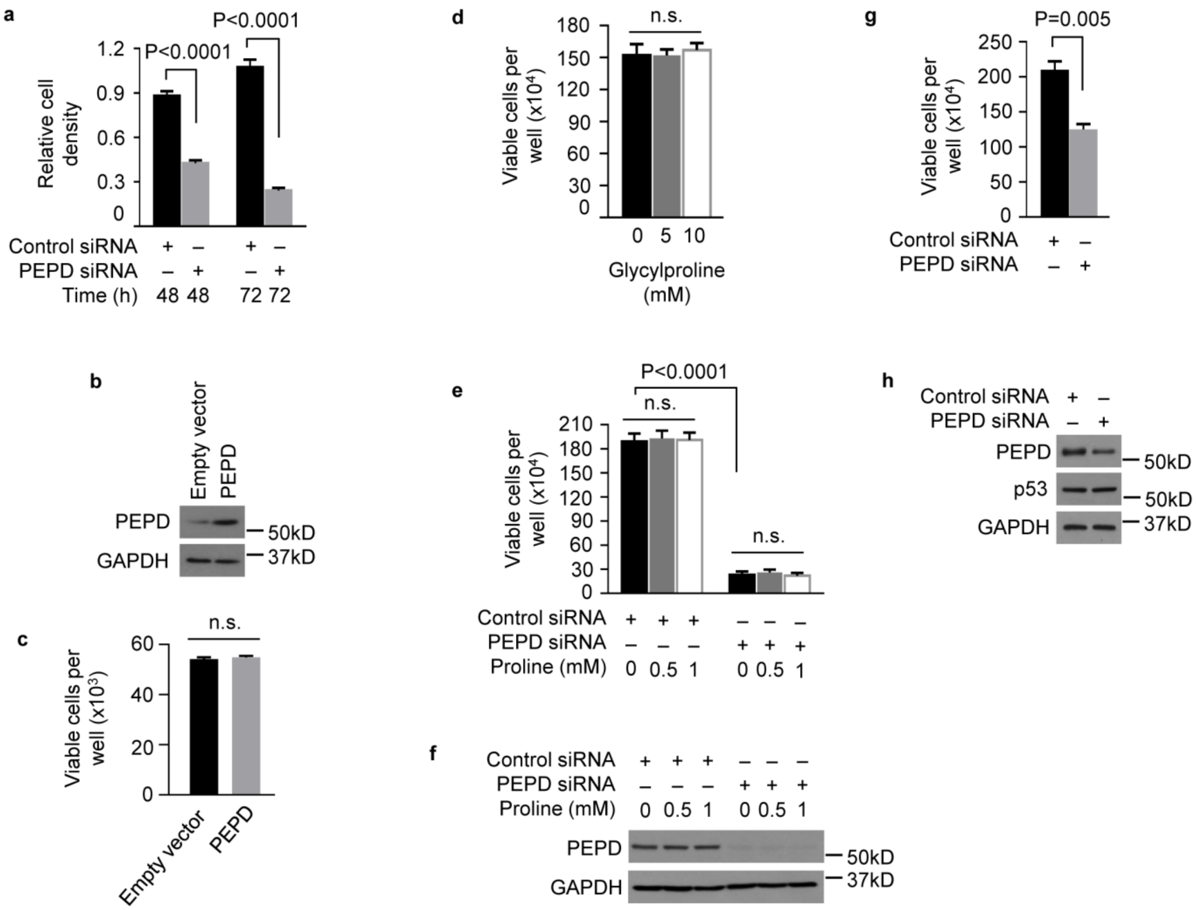

Supplementary Figure 4. The effect of PEPD on cell growth. (**a**) MTT assay of UM-UC-3 cell proliferation after siRNA treatment. Cells were grown in 96-well plates (2 x 10^3 cells/well) for 24 h before siRNA treatment. (**b, c**) IB analysis of PEPD and measurement of UM-UC-3 cell survival by trypan blue expulsion assay at 24 h after transfection with PEPD or EV. Cells were grown in 12-well plates (2 x 10^4 cells/well) for 24 h before plasmid transfection. (**d**) Measurement of UM-UC-3 cell survival by trypan blue expulsion assay after treatment with glycylproline (a PEPD substrate) or solvent for 72 h. Cells were grown in 6-well plates (5 x 10^4 cells/well) for 24 h before experiment. (**e, f**) Measurement of UM-UC-3 cell survival by trypan blue expulsion assay and IB analysis of PEPD after siRNA treatment for 24 h, followed by treatment with proline (a catalytic product of PEPD) or solvent for 72 h. Cells were grown in 6-well plates (5 x 10^4 cells/well) for 24 h before experiment. (**g, h**) Measurement of 32D cell survival by trypan blue expulsion assay and IB analysis of PEPD and p53 after siRNA treatment for 72 h. GAPDH was measured as a loading control. Cells were grown in 12-well plates (7.5 x 10^4 cells/well). Data are means ± s.d. (n=3); two-way ANOVA followed by Tukey multiple comparisons test in **a** and **e**; two-tailed t-test in **c** and **g**; one-way ANOVA in **d**; n.s., not significant.

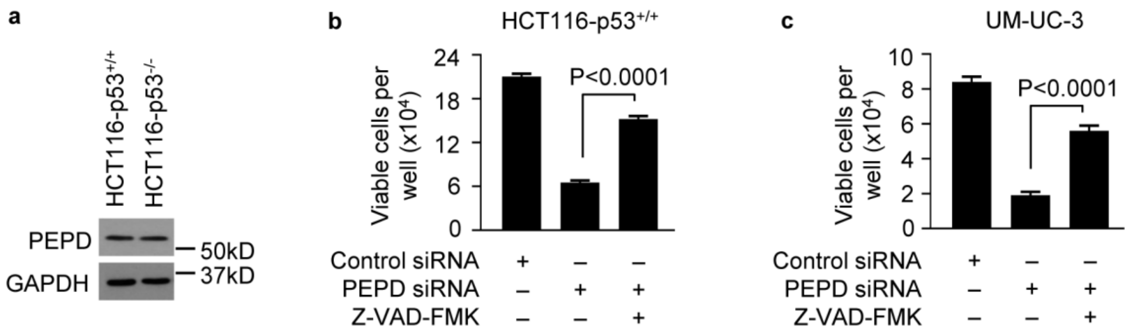

Supplementary Figure 5. No p53 effect on PEPD but PEPD knockdown induces apoptosis. (**a**) IB analysis of PEPD in cell lysates. (**b, c**) Measurement of cell viability after treatment with siRNA in the absence or presence of pan-caspase inhibitor Z-VAD-FMK for 72 h. Cells were grown in 48-well plate (5 x 10^3 cells/well for HCT116, and 6 x10^3 cells/well for UM-UC-3) for 24 h before experimental treatment. Z-VAD-FMK (20 μM) or solvent was added to the culture medium 4 h before siRNA transfection and then together with siRNA for 72 h. Data in **b** and **c** are means ± s.d. (n=3); two-way ANOVA followed by Tukey multiple comparisons test.

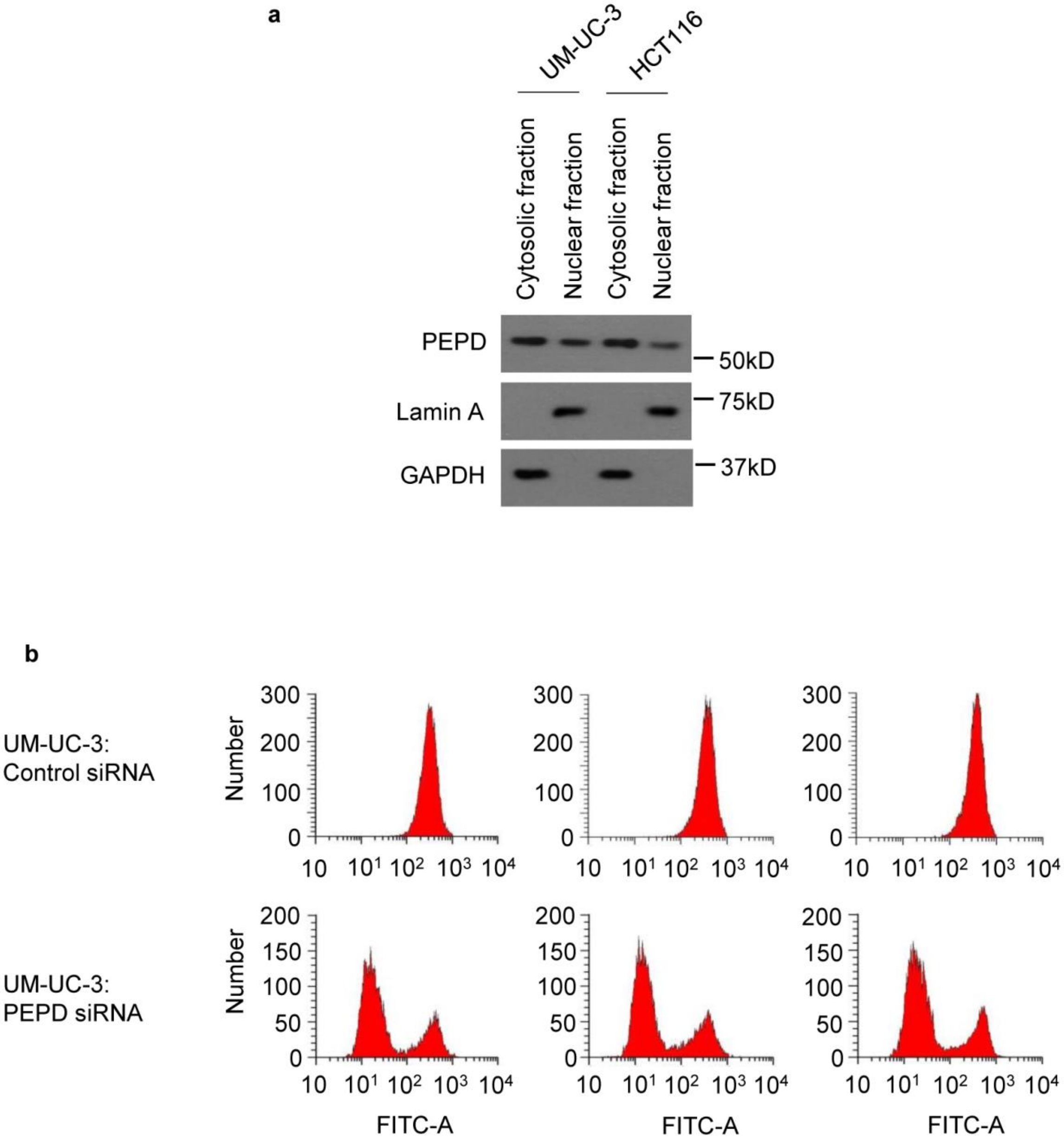

Supplementary Figure 6. Cytosolic/nuclear PEPD and the effect of PEPD siRNA on MMP. (**a**) IB analysis of PEPD in cytosolic fraction and nuclear fraction. Lamin A and GAPDH were measured to ensure the purity of the subcellular fractions. (**b**) Flow cytometry analysis of MMP in cells treated with siRNA for 72 h. Three independent experiments were performed to calculate the results shown in Figure 3d.

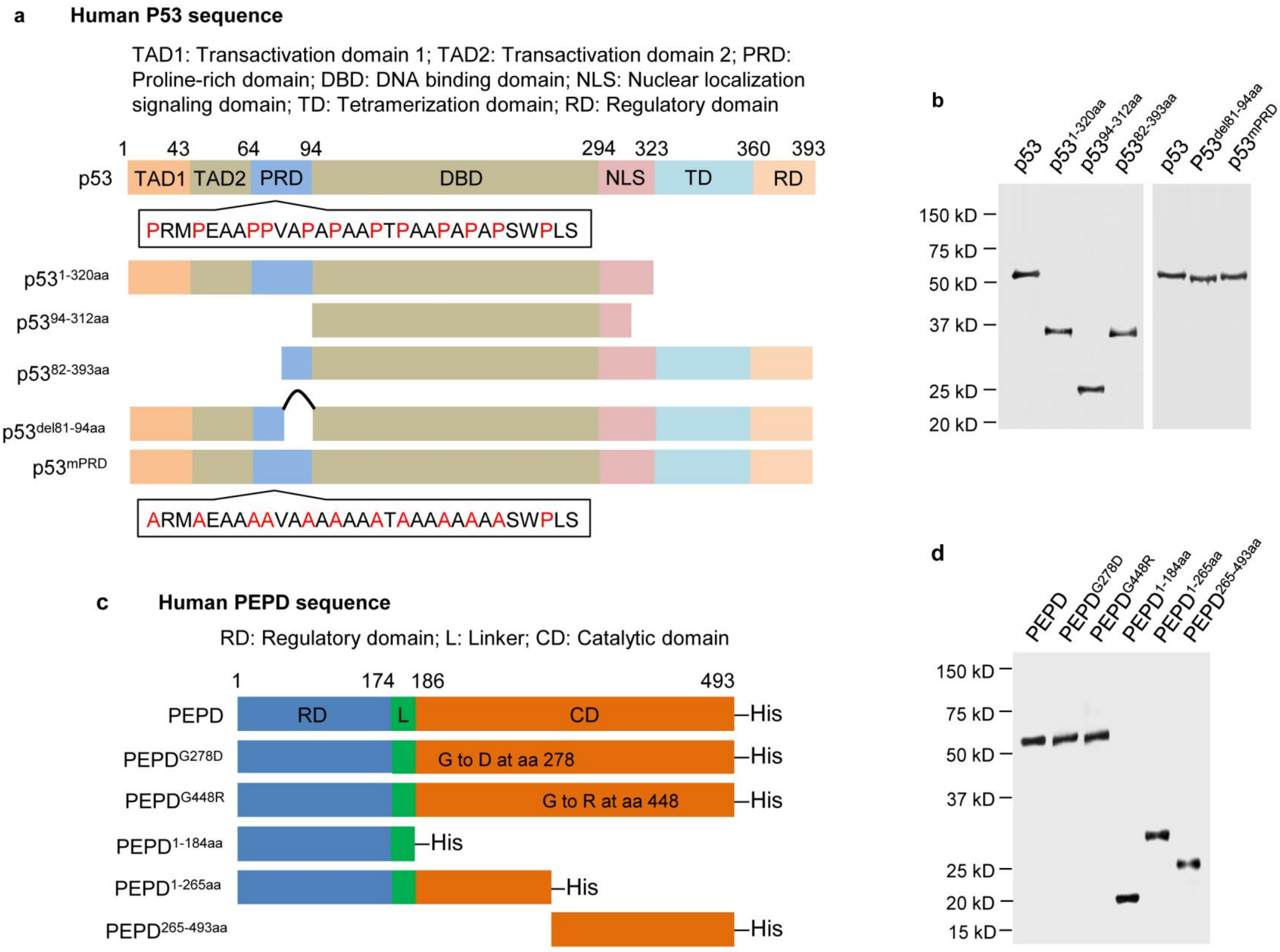

Supplementary Figure 7. p53 and PEPD as well as their mutants. (**a**) Amino acid sequences of human p53 and its mutants. (**b**) Bacteria-generated, affinity-purified human p53 and its mutants were analyzed by SDS-PAGE followed by silver staining. (**c**) Amino acid sequences of human PEPD and its mutants. (**d**) Bacteria-generated, affinity-purified recombinant human PEPD and its mutants were analyzed by SDS-PAGE, followed by silver staining.

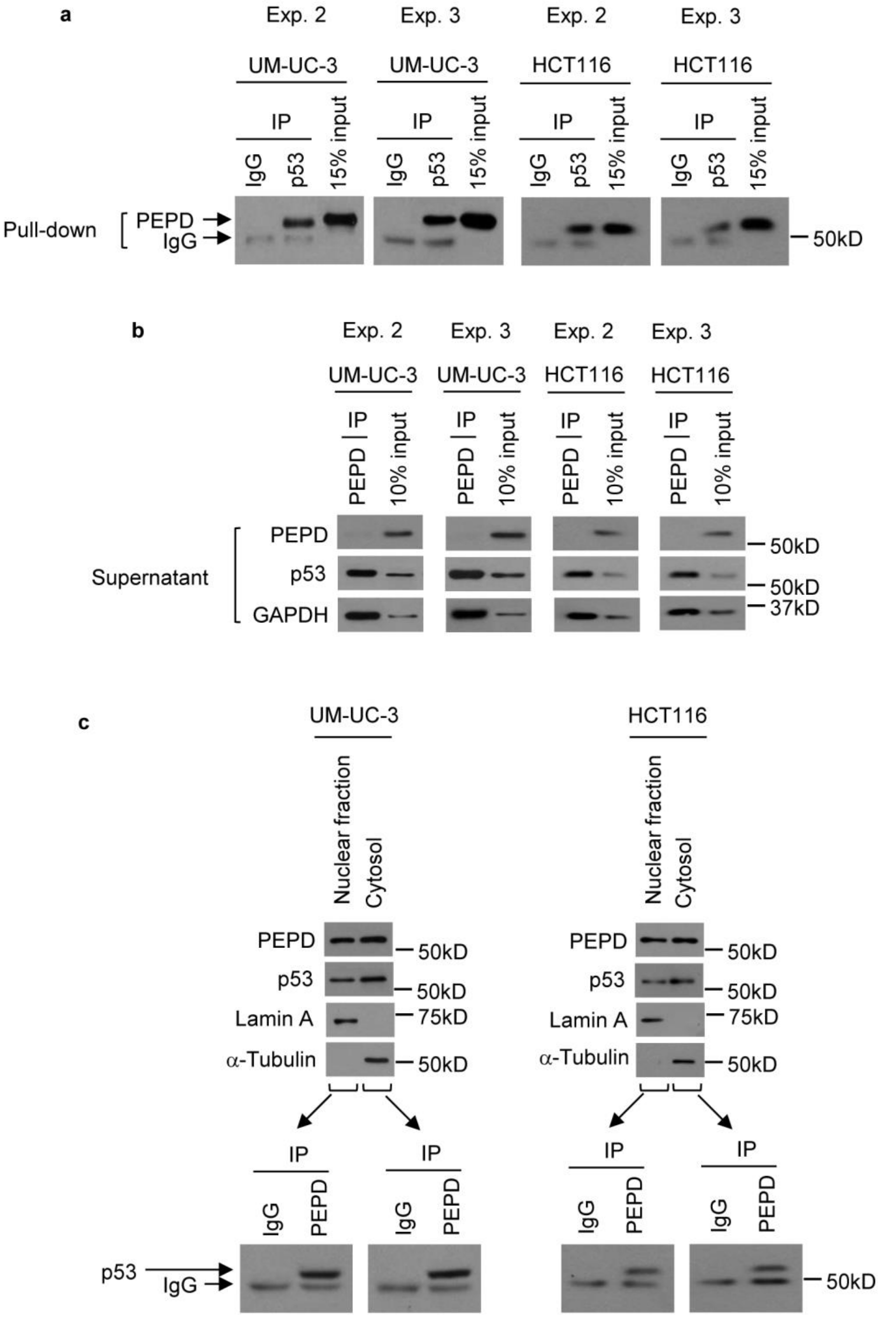

Supplementary Figure 8. PEPD association with p53 in cells. (**a**) IP-IB analysis of PEPD association with p53, with complete pull-down of p53 in the cell lysates, compared to the input. Two experiments (Exp. 2 and Exp. 3) are shown here. Exp. 1 is shown in Figure 5c. (**b**) IB analysis of p53 in the supernatant fraction, compared to the input, with complete pull-down of PEPD and PEPD-bound p53 in the cell lysates. Two experiments (Exp. 2 and Exp. 3) are shown here. Exp. 1 is shown in Figure 5e. (**c**) IB analysis of PEPD and p53 as well as IP-IB analysis of PEPD association with p53 in both nuclear fraction and cytosol. Lamin A and α-tubulin were measured to ensure the purity of subcellular fractions.

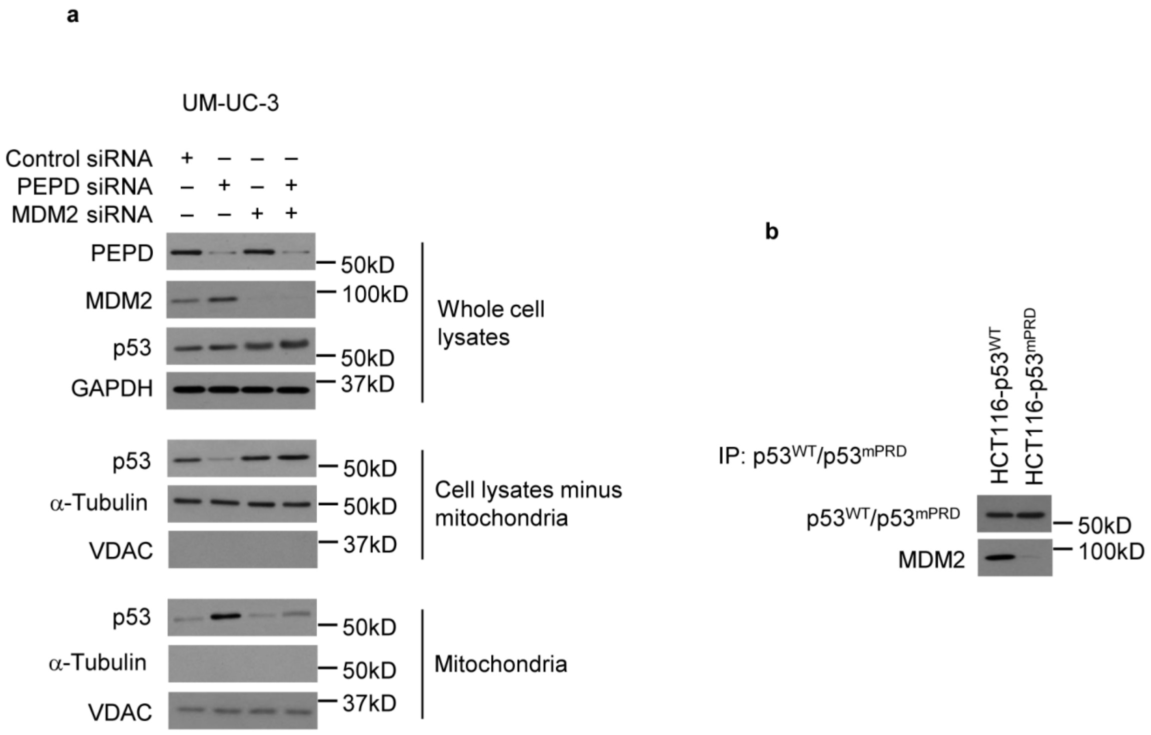

Supplementary Figure 9. PEPD blocks MDM2-mediated p53 movement to mitochondria. (**a**) IB analysis of p53 and other proteins in cell lysates and subcellular fractions. Cells were treated with control siRNA or MDM2 siRNA and 24 h later treated with control siRNA or PEPD siRNA for 48 h, from which whole cell lysates, cell lysates minus mitochondria or mitochondria fraction were prepared. MDM2 siRNA #2 (see Methods) was used in the experiment. GAPDH, α-tubulin and VDAC were measured to ensure the purity of subcellular fractions and as loading controls. (**b**) IP-IB analyses of MDM2 association with $p53^{WT}$ or $p53^{mPRD}$ in cell lysates. Cell lysates were subjected to pull-down of $p53^{WT}$ or $p53^{mPRD}$.

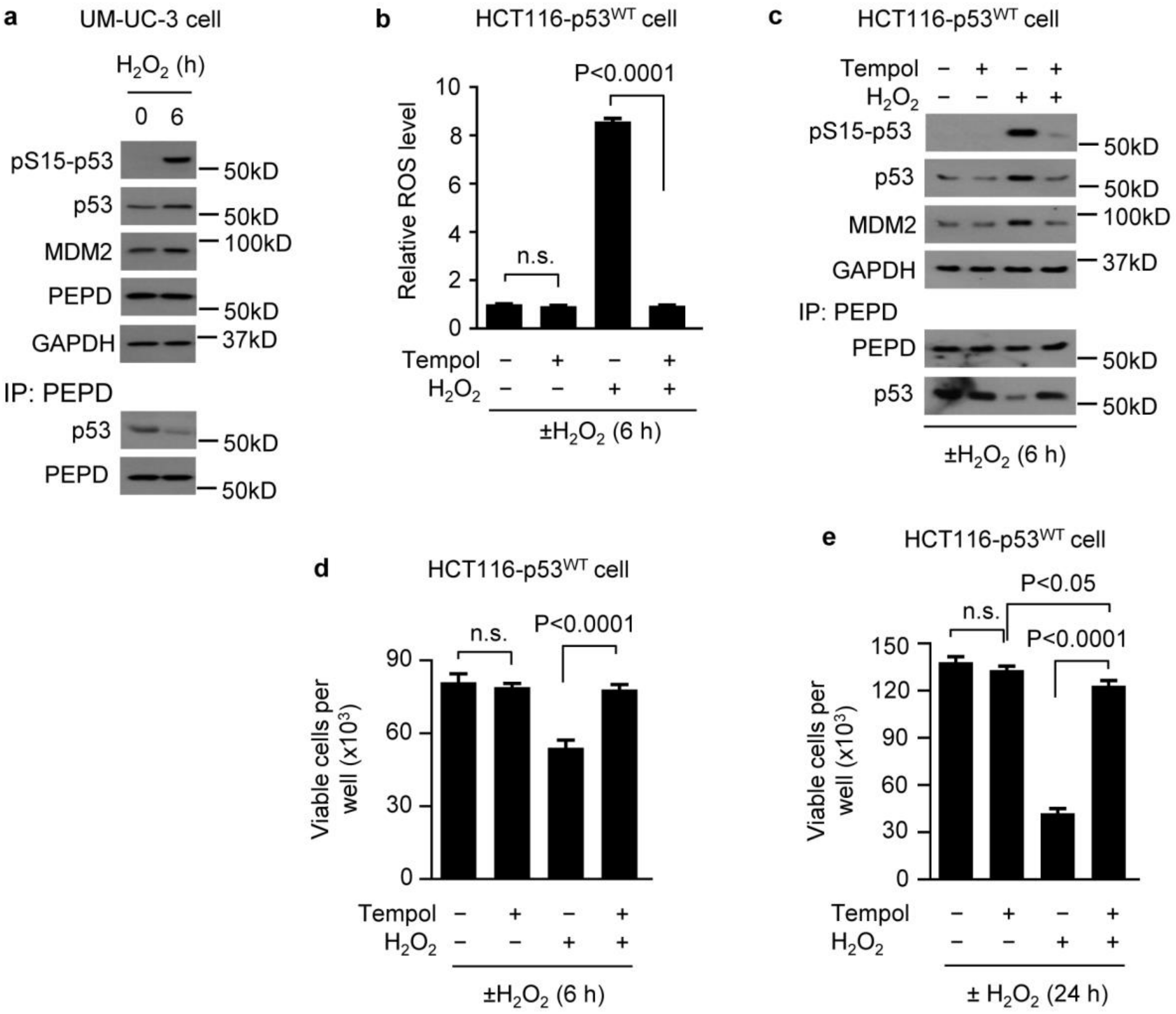

Supplementary Figure 10. p53 activation by H_2O_2 and the inhibitory effect of Tempol. (**a**) IB analysis of phosphor-p53, p53, MDM2 and PEPD as well as IP-IB analysis of p53 association with PEPD (PEPD pull-down) in control cells and cells treated with H_2O_2 for 6 h. GAPHD was measured as a loading control. (**b**) Relative ROS level in cells treated with solvent or H_2O_2 for 6 h, with or without Tempol pretreatment for 4 h. (**c**) IB and IP-IB analysis of various proteins in cells treated with solvent or H_2O_2 for 6 h, with or without Tempol pretreatment for 4 h. GAPDH was measured as a loading control. (**d, e**) Measurement of cell viability after treatment with H_2O_2 for 6 h or 24 h, with or without Tempol pretreatment for 4 h. In all experiments, cells were cultured in 12-well plates (4 x 10^4 cells/well) for 24 h before experimental treatment. H_2O_2 and Tempol were used at 400 μM and 0.5 mM, respectively. Data in **b**, **d** and **e** are means ± s.d. (n=3); two-way ANOVA followed by Tukey multiple comparisons test; n.s., not significant.

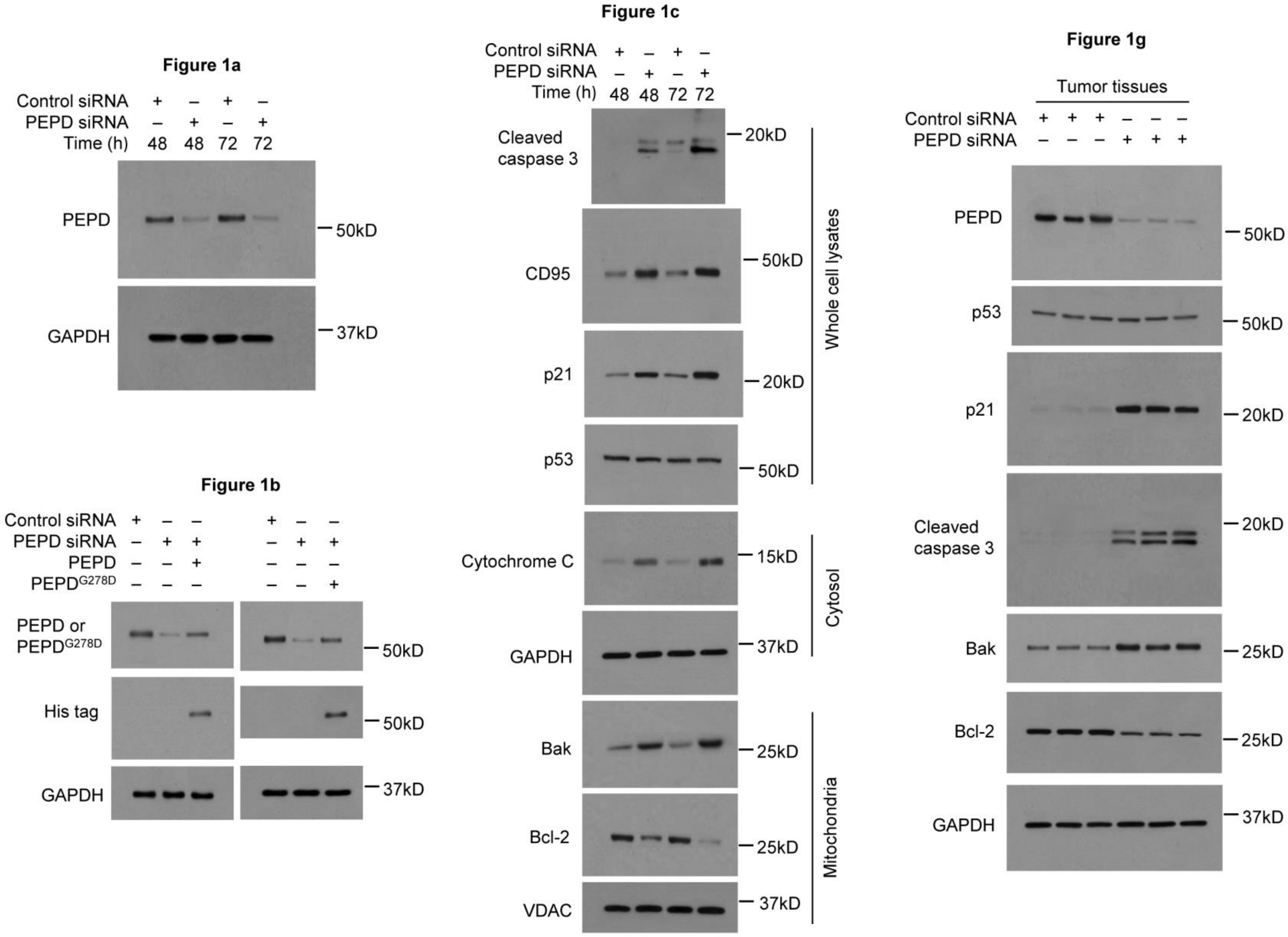

Supplementary Figure 11. Uncropped IB images for results shown in Figure 1.

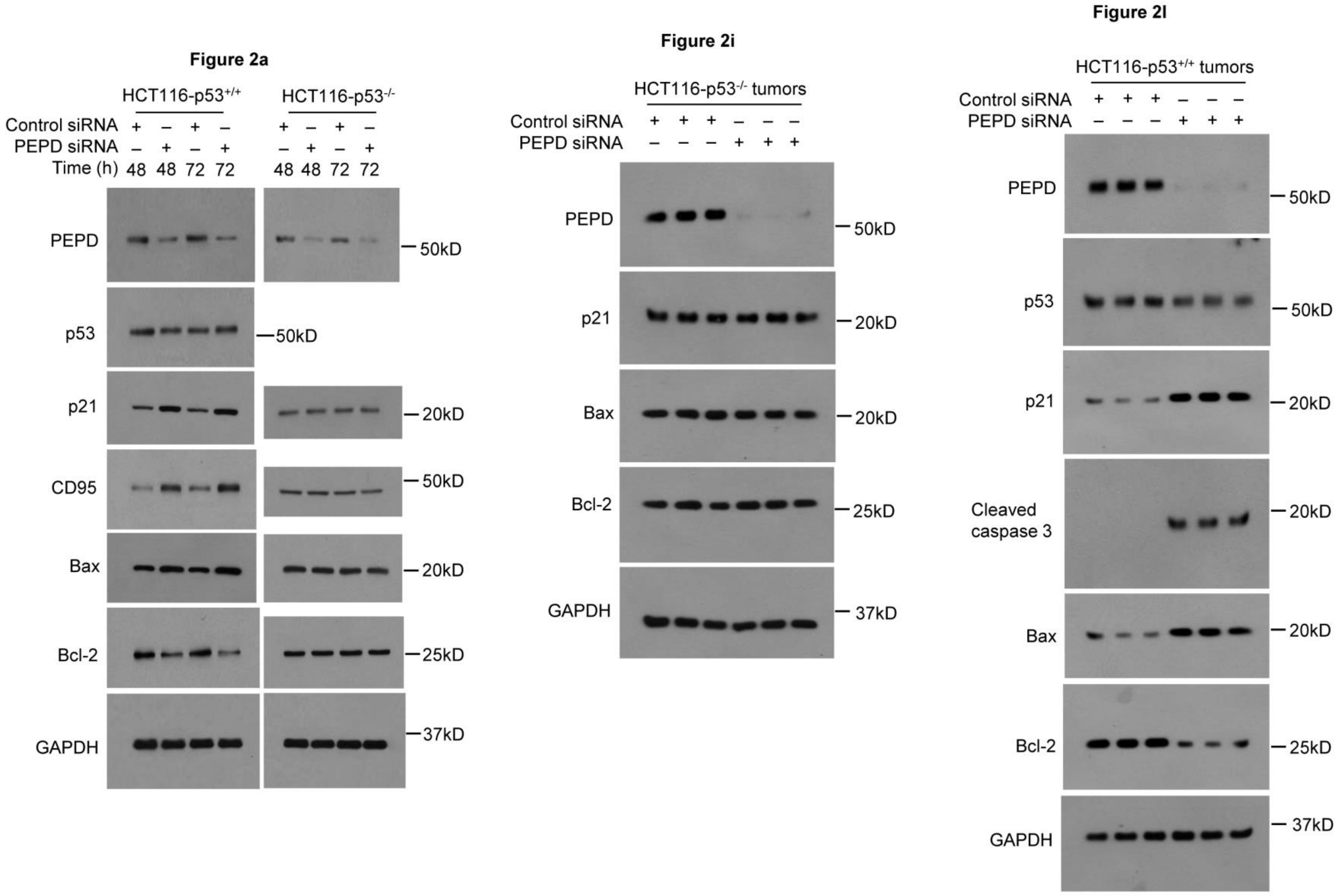

Supplementary Figure 12. Uncropped IB images for results shown in Figure 2.

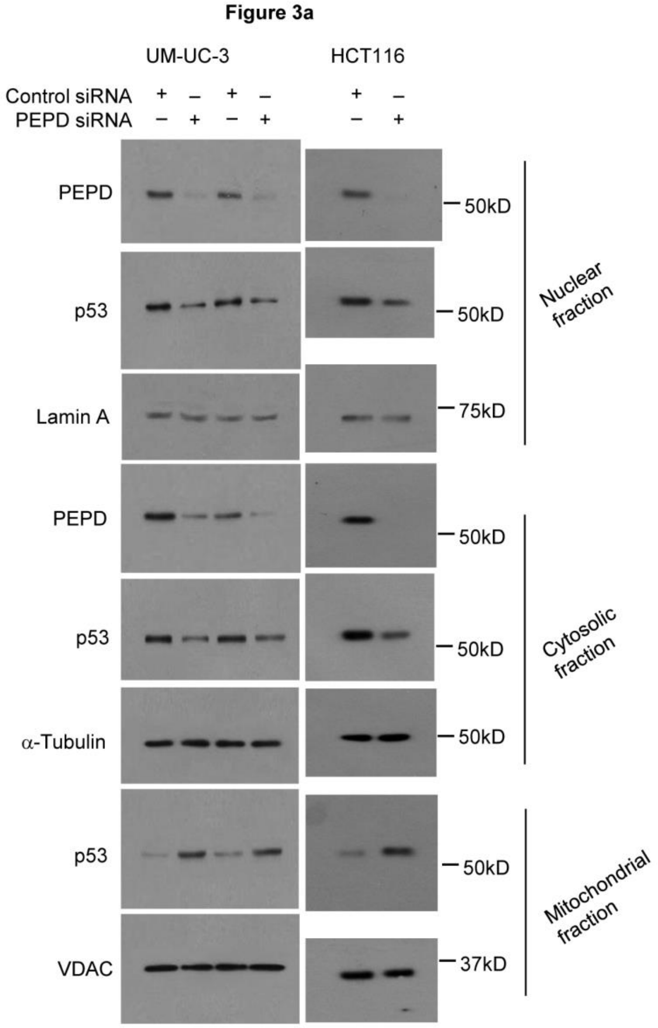

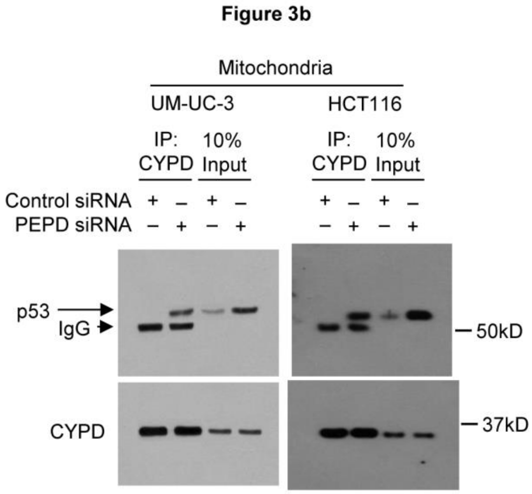

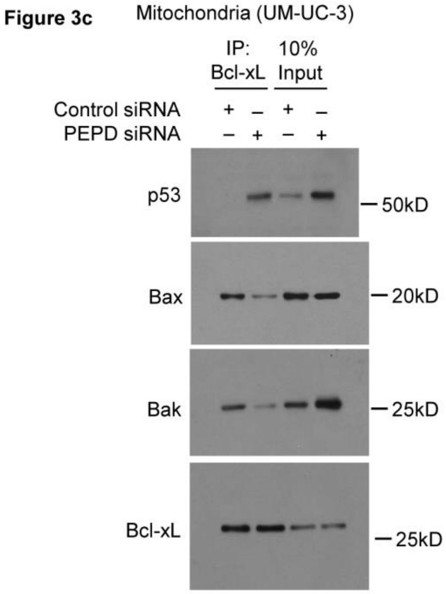

Supplementary Figure 13. Uncropped IB images for results shown in Figure 3.

Figure 4d

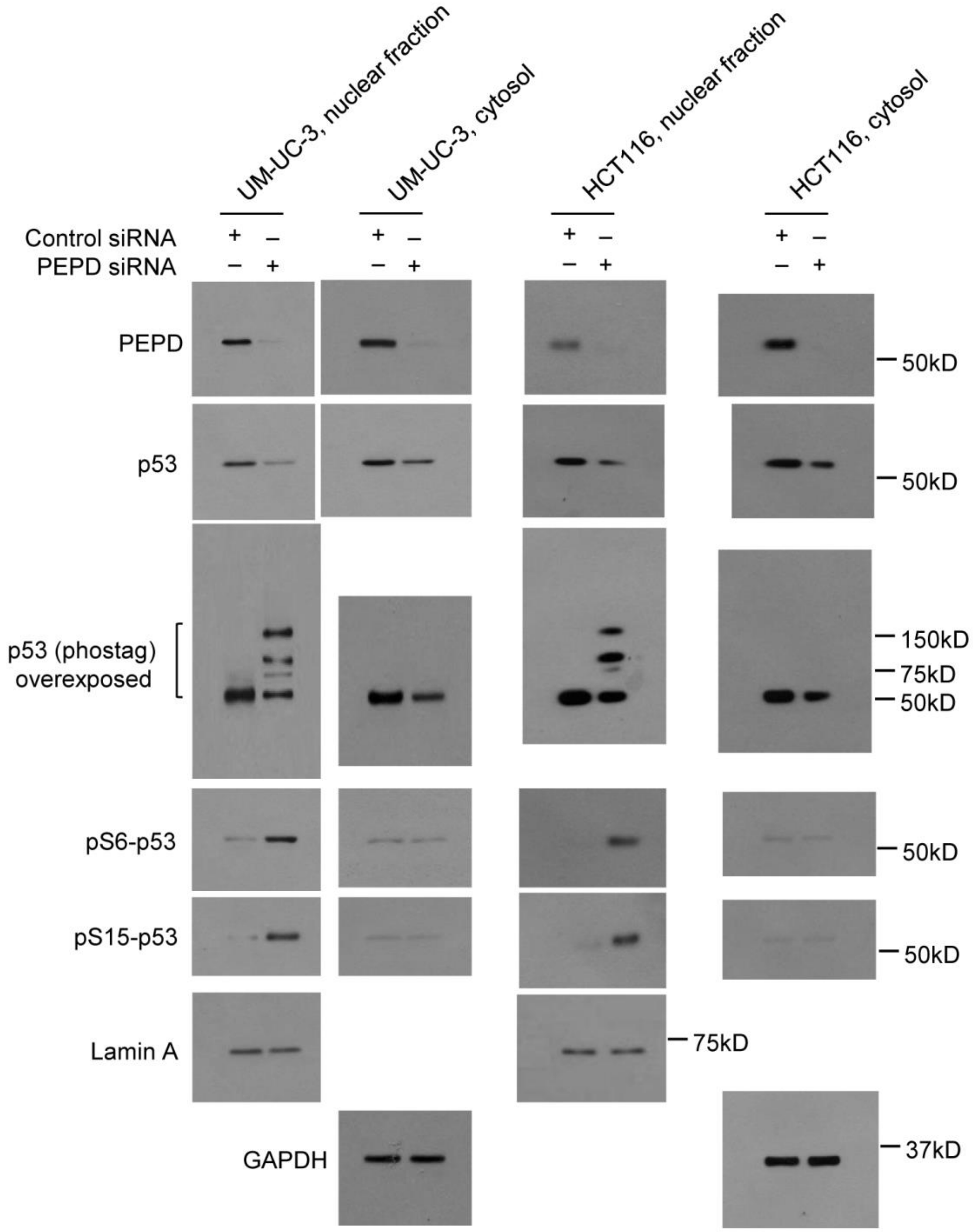

Supplementary Figure 14. Uncropped IB images for results shown in Figure 4.

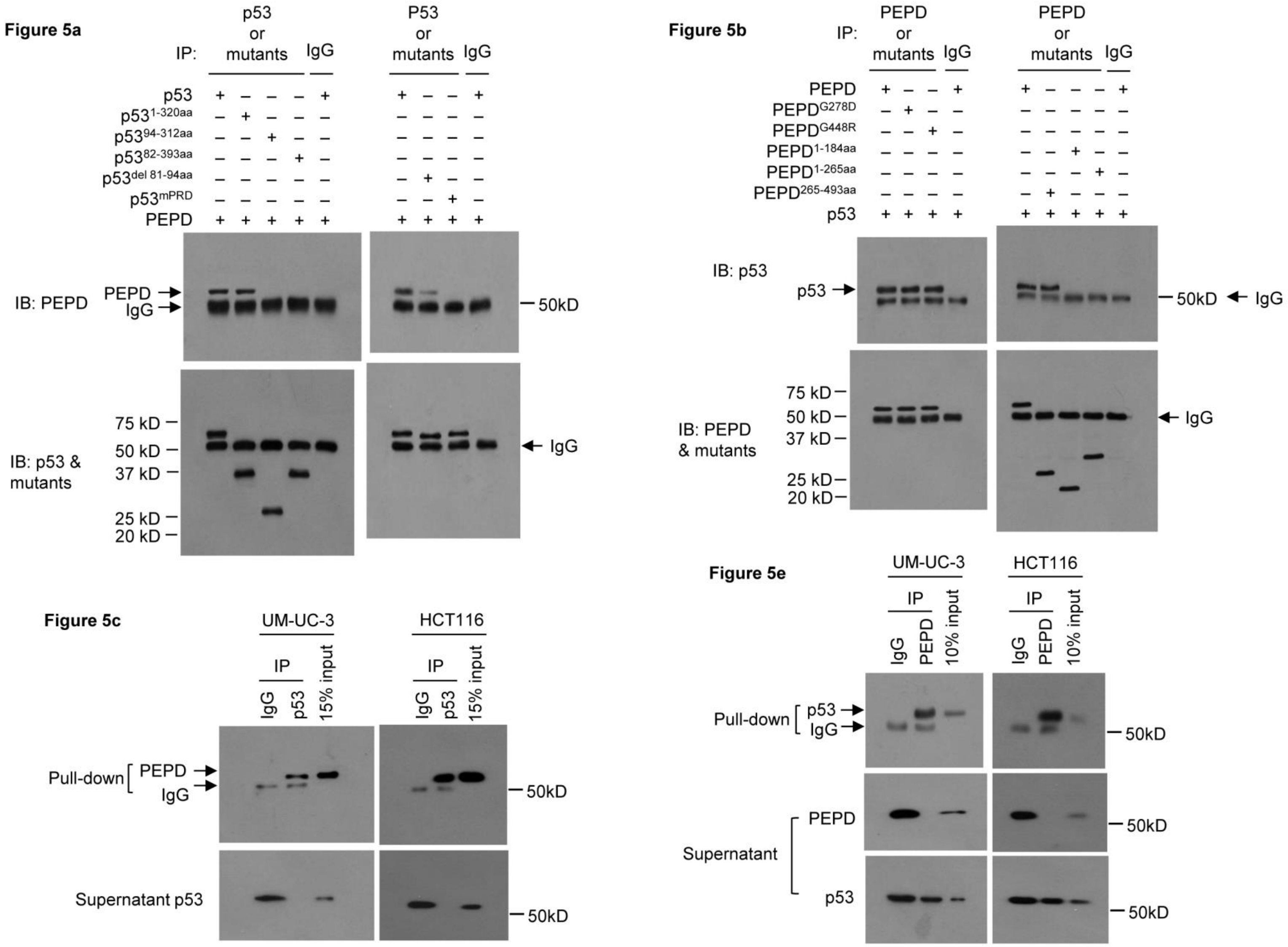

Supplementary Figure 15. Uncropped IB images for results shown in Figure 5.

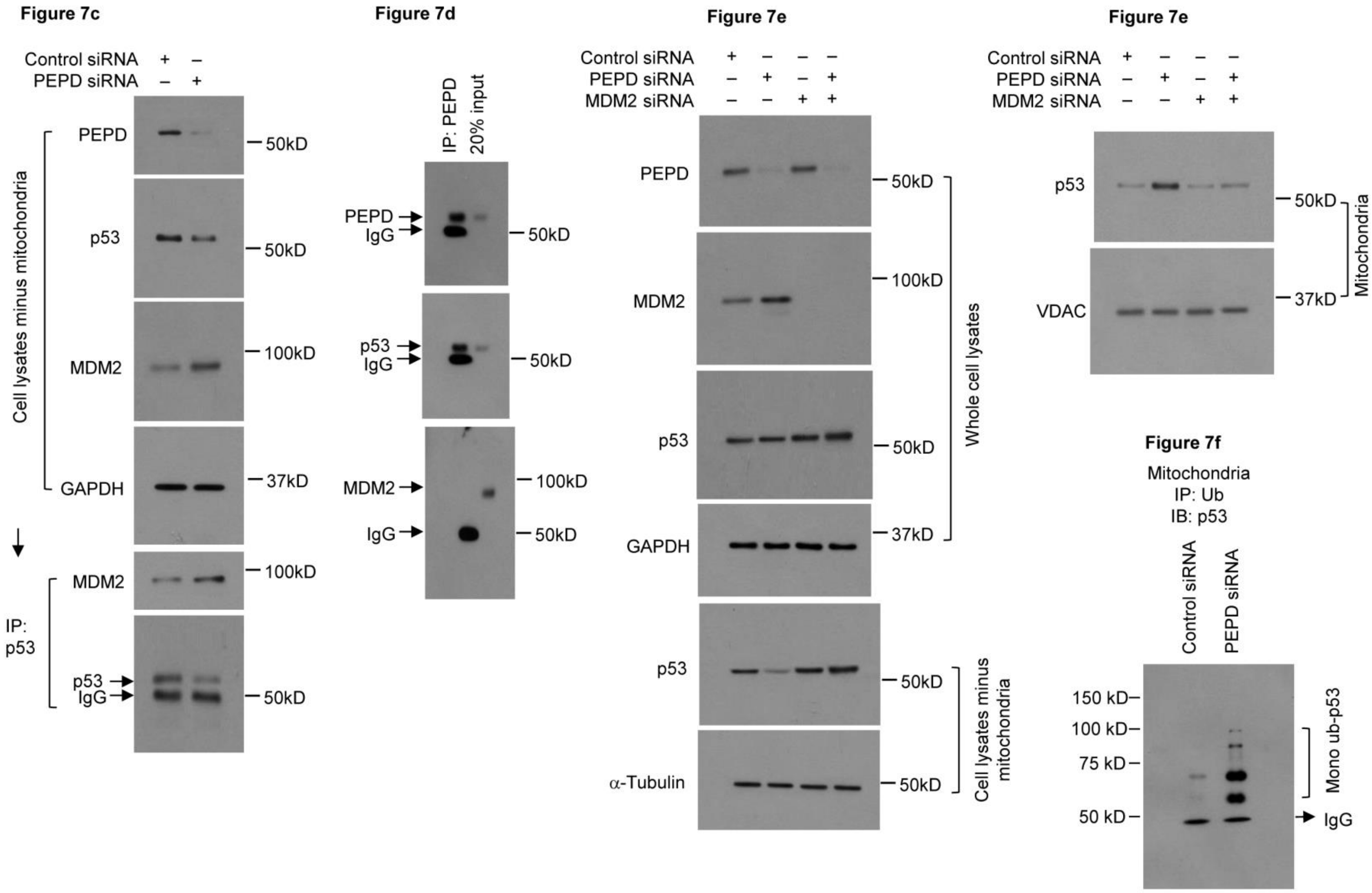

Supplementary Figure 16. Uncropped IB images for results shown in Figure 7.

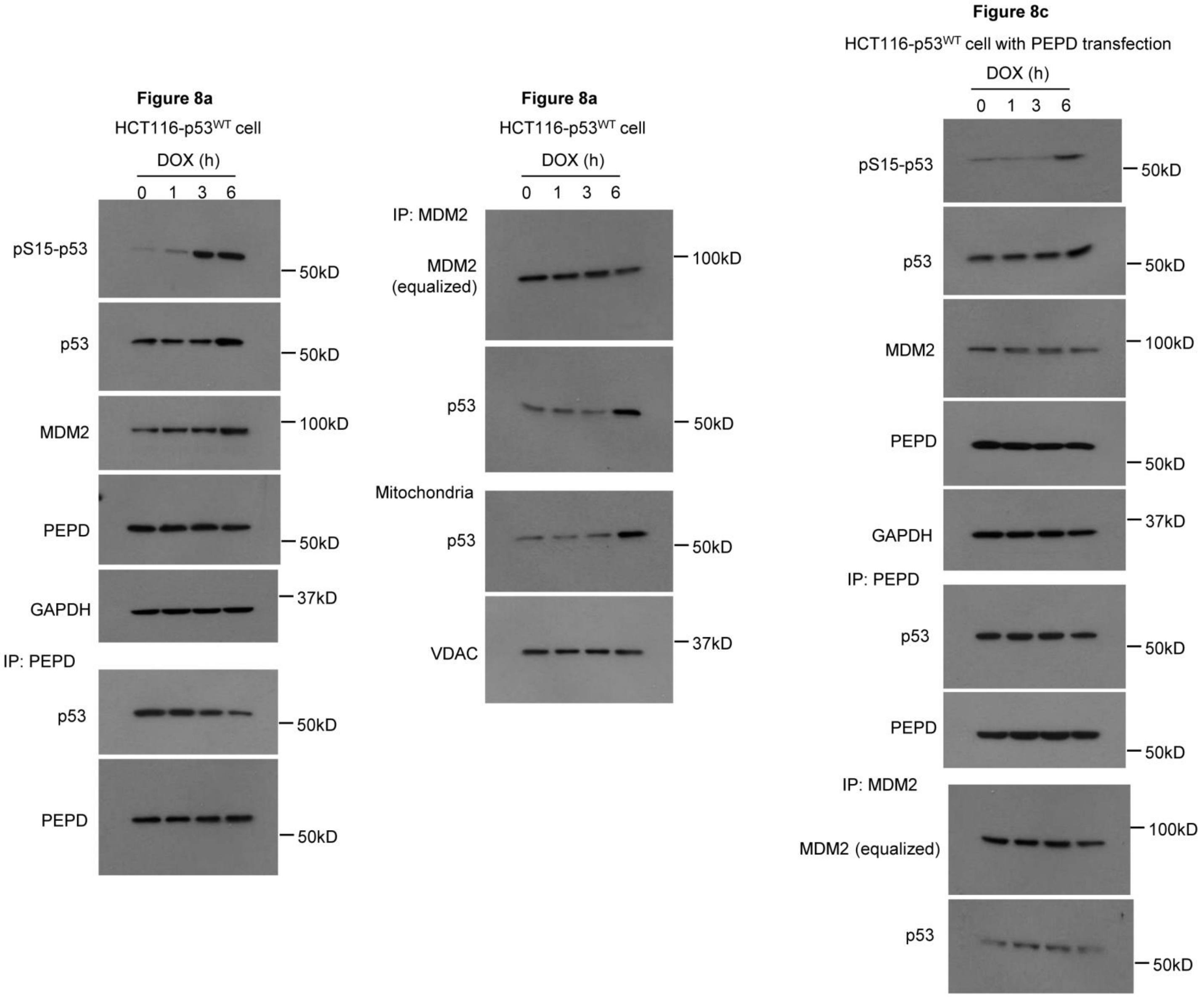

Supplementary Figure 17. Uncropped IB images for results shown in Figure 8.

Figure 8e

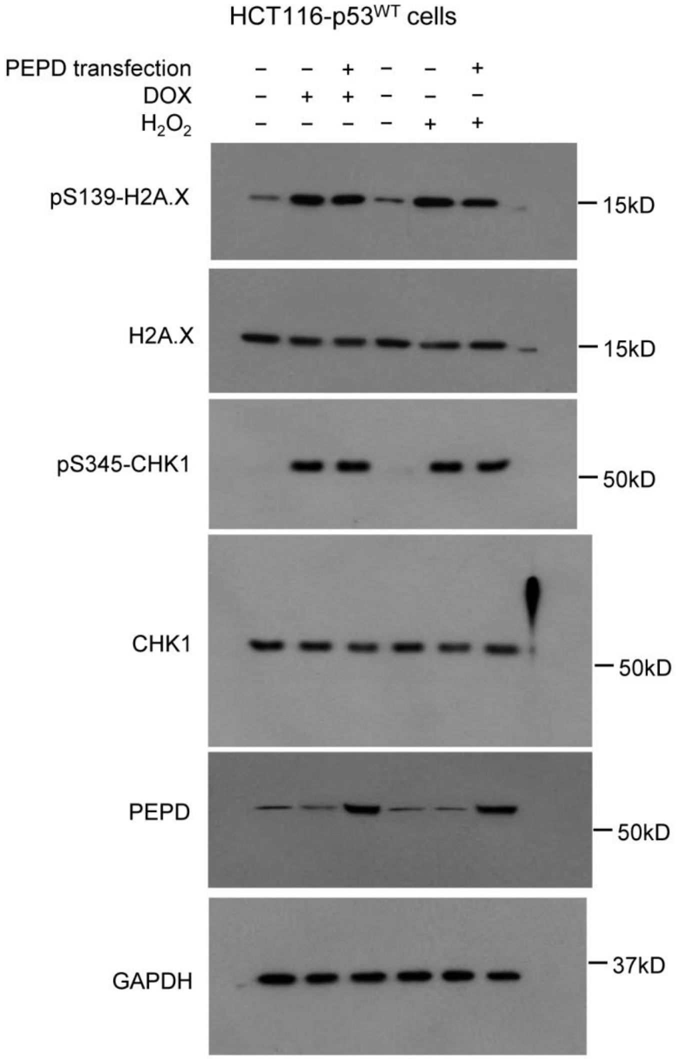

Supplementary Figure 18. Uncropped IB images for results shown in Figure 8.

Figure 9b

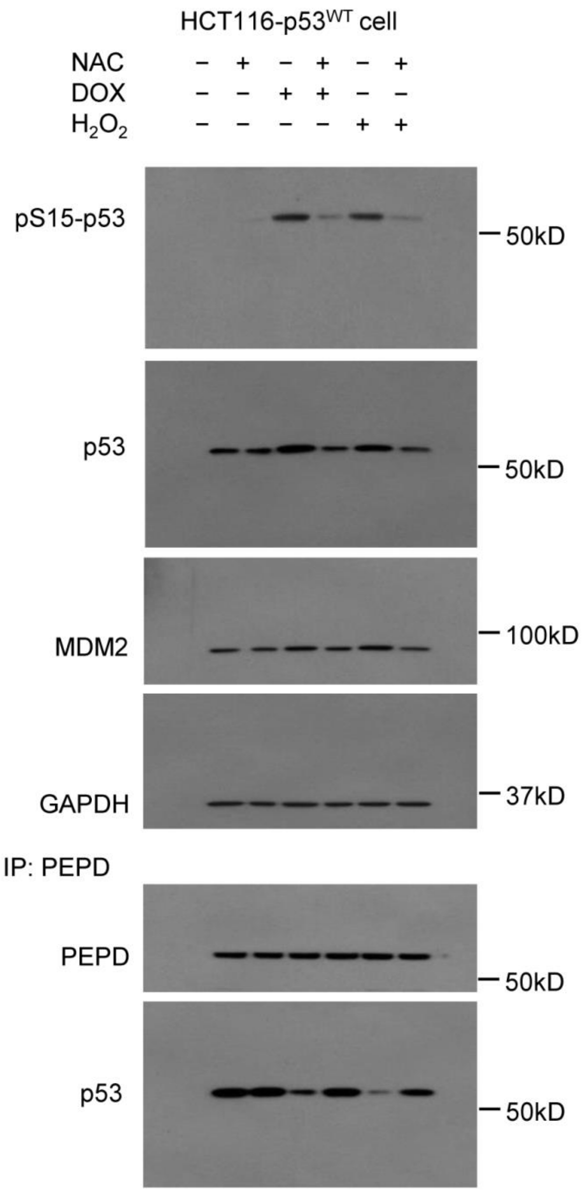

Supplementary Figure 19. Uncropped IB images for results shown in Figure 9.
