# Supplementary material for: PEPD is a pivotal regulator of p53 tumor suppressor
Source: Nat Commun. 2017 Dec 12;8:2052. doi: 10.1038/s41467-017-02097-9 (PMC5727116; doi:10.1038/s41467-017-02097-9)
Supplement: Supplementary file 2 — Peer Review File [file 41467_2017_2097_MOESM2_ESM.pdf]

Reviewer #1 :
(Remarks to the Author):

The manuscript by Yang et al. describes a potential novel link between p53 and PEPD, Peptidase enzyme. They found that PEPD suppresses p53 function by directly binding to p53 in both nucleus and cytoplasm, which inhibits both transcription-dependent and –independent activities of p53. They further find that PEPD-mediated inhibition of p53 function is essential for cell survival in response to stress and promotes tumor growth. Based on these findings, the authors suggest that PEPD promotes tumor progression by decreasing the efficacy of p53 to direct appropriate responses to DNA damage. Their findings include:

(1) PEPD loss leads to cell death and tumor regression in UM-UC3 cells in vitro.
(2) PEPD protects cells against p53-dependent DNA damage-induced apoptosis.
(3) PEPD inhibits transcription-independent cell-killing function of p53.
(4) PEPD inhibits p53 transcriptional activity.
(5) PEPD directly binds to the proline-rich domain in p53.
(6) PEPD prevent MDM2-directed mitochondrial translocation of p53.
(7) ROS generated by stress frees p53 from PEPD binding, to enable tp53 activation.
Overall, the work is well performed and well described. The main missing part of the puzzle is how PEPD pathway leads to a modulation of p53 activity, and how PEPD attenuates DNA damage-induced p53 activation since p53 protein is post-translationally regulated. In addition, it is confusingly presented how PEPD binding to p53 affects p53-dependent transactivation. They present numerous sub-related stories, each of which is of interest, but these need to be more mechanically connected to get an overview of the significance of the PEPD-mediated p53 regulation. In addition ROS connection seems preliminary. To increase the relevance to cancer biology, it would be informative to investigate this unexpected relationship between p53 and PEPD by using a PEPD-null mouse model.
Other issues:
- PEPD binds to mutant p53s that lost transactivation.
- UM-U3 cell was mainly used for most of the studies.

Reviewer #2:
(Remarks to the Author):

The study by Yang et al addresses a very important question of regulation of the tumor suppressor p53. It is a very original, deep and thorough study showing that PEPD binds to and suppress p53, at the same time keeping p53 available for a very fast response to ROS-inducing stress conditions. This is technically a very sound paper, the results of experiments are very convincing.
As a novel findings usually do, these results open several new avenues of research. This study will be interesting for researchers working in cancer field, but not only those.
Further studies in this direction might cause a paradigm shift, that is, to question the idea that p53 is inhibited mainly by mdm2/mdmX in cancer.

I think this study is excellent and should be published in Nat Communic, with some minor changes:

1. In Fig 5g, is it immunoprecipitation of endogenous PEPD or pull-down by exogenous PEPD? Please clarify
2. page 11, it's auto-regulatory loop, not antiregulatory loop

It would be interesting to know, whether PEPD controls p53 also in normal cells and is the cell death induced by PEPD depletion could be prevented by ZVAD - thus, is it mainly apoptotic?

Reviewer #3 :
(Remarks to the Author):

The Authors provide original, well documented data that prolidase (PEPD) is an inhibitor of P53 protein through complex formation and that the PEPD ability is independent on the enzymatic activity of PEPD. They presented potential mechanism of PEPD-dependent regulation of survival/apoptosis in cancer cell lines as well as PEPD-dependent tumor growth/inhibition in animal model. The Authors suggest that underlying mechanism of PEPD-dependent inhibition of apoptosis is formation of complex with P53 and that the complex is sensitive to reactive oxygen species (ROS) that release P53 from complex and facilitate apoptosis. This is elegant study providing several arguments to support the conclusion that PEPD facilitate cell survival and stores p53 for stress response. Moreover, convincing data were provided that PEPD binds to p53, which inhibits phosphorylation of nuclear p53 and MDM2-mediated mitochondrial translocation of nuclear and cytoplasmic p53. The findings are of great importance to understand the role of prolidase in biology of cancer and are particularly important for development of new tools for cancer treatment.

Reviewer 1

Point 1: "Overall, the work is well performed and well described. The main missing part of the puzzle is how PEPD pathway leads to a modulation of p53 activity, and how PEPD attenuates DNA damage-induced p53 activation since p53 protein is post-translationally regulated. In addition, it is confusingly presented how PEPD binding to p53 affects p53-dependent transactivation. They present numerous sub-related stories, each of which is of interest, but these need to be more mechanically connected to get an overview of the significance of the PEPD-mediated p53 regulation."

Response: Different aspects of PEPD interaction with p53 are tied together in the Discussion and in Fig. 10. In view of this comment, we have revised the relevant text and tagged key statements with specific figures, to make it clearer and more understandable.

In the first paragraph under Discussion in the revised manuscript (pages 14-15), the text now reads: "*We show that the C-terminal sequence of PEPD binds to the PRD in p53 (Fig. 5), which allows PEPD to accomplish two important tasks: 1) to prevent nuclear p53 phosphorylation in its transactivation domain (Fig. 4) and to reduce free nuclear p53 level (Fig. 5), leading to inhibition of p53 trans-activation and trans-suppression activities (Figs 1, 2, 4), and 2) to prevent mitochondrial translocation of nuclear and cytosolic p53 by preventing p53 from binding to MDM2 (Figs 3, 6, 7). PEPD sequesters more than 50% of cellular p53 under normal conditions (Fig. 5). PEPD modulates p53 without requiring its enzymatic activity (Fig. 1)……* We show that stress signals, using H_2O_2 and DOX as examples, must free p53 from PEPD, via ROS, in order to achieve robust p53 activation and that the p53-PEPD complex is designed to rapidly mobilize a large amount of pre-synthesized p53 to counter stress (Figs 8-9)."

In the fourth paragraph under Discussion in the revised manuscript (page 16), the text now reads: "*Our study also reveals a previously unrecognized anticancer mechanism of DOX. It is currently widely accepted that DOX-induced DNA damage causes activation of certain protein kinases which in turn activate and stabilize p53. However, we show that the key step in DOX-induced p53 activation is the disruption of p53 association with PEPD via ROS. This is also true for p53 activation and cell killing by H_2O_2.*" In addition, we have added new result (CHK1

phosphorylation in Fig. 8e) to further show that attenuation of DNA-damage-induced p53 activation by PEPD overexpression is not due to inhibition of DNA damage, rather inhibition of p53 separation from PEPD.

Point 2: “ROS connection seems preliminary.”

Response: New results have been added. In the previous manuscript, we showed that treatment of cells with DOX or H_2O_2 causes marked increase in ROS, p53 separation from PEPD, p53 activation, and cell death, and that N-acetylcysteine, which effectively quenches ROS, prevents p53 separation, p53 activation and cell death (Fig. 9), all of which are included in the revised manuscript. New results have been added to show that anther ROS quencher Tempol, which is structurally very different from N-acetylcysteine, is also highly effective in preventing the stress signal from freeing p53 from PEPD, with inhibition of p53 activation and cell death. Due to space restraint, the new results are presented in Supplementary Figs 10b-e.

Point 3: “To increase the relevance to cancer biology, it would be informative to investigate this unexpected relationship between p53 and PEPD by using a PEPD-null mouse model.”

Response: PEPD knockout is lethal to both cancer cells and normal cells as shown in Supplementary Figs 1-3 and therefore is most likely embryonically lethal. Although conditional PEPD knockout may be feasible, data from such mice are likely very difficult to interpret, because PEPD knockout also means total loss of PEPD enzymatic activity, which is known to cause multi-organ abnormalities due to defective collagen metabolism, known as PEPD deficiency, as described in the first paragraph in the Introduction. It will be a major undertaking to sort out which phenotypical changes result from p53 activation and which phenotypical changes result from defective collagen metabolism, which will require a new study. This is now mentioned in the revised manuscript (page 15, lines 3-5).

In the meantime, we have added new data to show that PEPD knockdown-induced tumor inhibition is completely p53-dependent. We generated subcutaneous tumors in nude mice by inoculating syngeneic human colon cancer HCT116-p53$^{+/+}$ cells and HCT116-p53$^{-/-}$ cells. Intratumor injection of PEPD siRNA lead to PEPD knockdown in both types of tumors, but HCT116-p53$^{-/-}$ tumor growth was not affected by PEPD knockdown at all, whereas PEPD knockdown caused marked inhibition of HCT116-p53$^{+/+}$ tumors with activation of p53 targets (Fig. 2g-l). These results further show the importance of the PEPD-p53 system is cancer cells.

Point 4: “PEPD binds to mutant p53s that lost transactivation?”

Response: Our results in Fig. 5a suggest that p53 mutants with intact PRD may bind to PEPD. We have not evaluated various p53 mutants to confirm their binding to PEPD, but we have added a sentence in the revised manuscript (page 8, line 4 from bottom) to indicate this possibility: “Our results also suggest that PEPD may bind to certain p53 mutants.”

Point 5: “UM-UC-3 cell was mainly used for most of the studies.”

Response: UM-UC-3 cells were used in experiments presented in Figs 1-5 and 7 as well as Supplementary Figs 1, 4-6, and 8-10. However, HCT116 cells (WT p53 and/or p53 null) were also used in experiments presented in Figs 2-6, 8 and 9, as well as Supplementary Figs 5, 6, and 8-10. We saw no difference in PEPD modulation of p53 between UM-UC-3 cells and HCT116 cells (WT p53). Therefore, in a few experiments, only one cell line was used. We also presented results from normal human bladder epithelial cells in Supplementary Fig. 2 and immortalized human bladder epithelial cells in Supplementary Fig. 3. Moreover, in addition to presenting UM-UC-3 tumor data in Figs 1e-g, we have now added HCT116-p53$^{+/+}$ tumor data and HCT116-p53$^{-/-}$ tumor data in Figs 2g-l).

Review 2

Point 1: "In Fig 5g, is it immunoprecipitation of endogenous PEPD or pull-down by exogenous PEPD? Please clarify".

Response: Yes, it is immunoprecipation of endogenous PEPD, as indicated in the figure legend.

Point 2: "page 11, it's auto-regulatory loop, not antiregulatory loop".

Response: Thank you. It has been revised as recommended. Please see page 11, line 6, and page 15, line 9.

Point 3: "It would be interesting to know, whether PEPD controls p53 also in normal cells and is the cell death induced by PEPD depletion could be prevented by ZVAD - thus, is it mainly apoptotic?"

Response: We have added new results to show that PEPD knockout by CRISPR/Cas9 kills both normal human bladder epithelial cells (Supplementary Fig. 2) and immortalized human bladder epithelial cells (UROtsa) (Supplementary Fig. 3). Please note that the colors of the images in Supplementary Figs 2-3 are different from that in Supplementary Fig. 1; the new results were obtained using a new microscope. Because all cells died quickly in the above experiments, we were not able to measure p53 activation. We have also added new results to show that pan-caspase inhibitor Z-VAD-FMK rescues cells against PEPD knockdown, increasing the survival of HCT116 cells (WT p53) and UM-UC-3 cells treated by PEPD siRNA (72 h) by 233.9-296.7% (Supplementary Figs 5b-c). However, Z-VAD-FMK did not completely prevent cell death despite using optimal concentration, as shown in Supplementary Figs 5b-c. It is possible that some of the cells die through p53-dependent but non-apoptotic mechanisms.

Reviewer 3

There is no critique from this reviewer. We appreciate your enthusiasm and positive response.

Think you again for considering this work for Nature Communications. We look forward to receiving your feedback soon.

Yuesheng Zhang, MD, PhD
Professor of Oncology

Reviewer #1 (Remarks to the Author):

The revised manuscript is quite satisfactory. The critiques were well responded. No further concerns from this reviewer.

Reviewer #2 (Remarks to the Author):

The authors have addressed all the points raised by me, thus I conclude that the manuscript is ready to be published in Nature Communications
